# Metabolic remodeling in cardiac hypertrophy and heart failure with reduced ejection fraction occurs independent of transcription factor EB in mice

**DOI:** 10.3389/fcvm.2023.1323760

**Published:** 2024-01-08

**Authors:** Niklas Dörmann, Elke Hammer, Karlotta Struckmann, Julia Rüdebusch, Kirsten Bartels, Kristin Wenzel, Julia Schulz, Stefan Gross, Stefan Schwanz, Elisa Martin, Britta Fielitz, Cristina Pablo Tortola, Alexander Hahn, Alexander Benkner, Uwe Völker, Stephan B. Felix, Jens Fielitz

**Affiliations:** ^1^ DZHK (German Center for Cardiovascular Research), Partner Site Greifswald, Greifswald, Germany; ^2^ Interfaculty Institute for Genetics and Functional Genomics, University Medicine Greifswald, Greifswald, Germany; ^3^ Department of Internal Medicine B, Cardiology, University Medicine Greifswald, Greifswald, Germany; ^4^ Experimental and Clinical Research Center, Max Delbrück Center for Molecular Medicine in the Helmholtz Association, Charité Universitätsmedizin Berlin, Berlin, Germany

**Keywords:** TFEB, left ventricular hypertrophy, heart failure with reduced ejection fraction, metabolic remodeling, fatty acid oxidation, transverse aortic constriction

## Abstract

**Background:**

A metabolic shift from fatty acid (FAO) to glucose oxidation (GO) occurs during cardiac hypertrophy (LVH) and heart failure with reduced ejection fraction (HFrEF), which is mediated by PGC-1α and PPARα. While the transcription factor EB (TFEB) regulates the expression of both *PPARGC1A*/PGC-1α and *PPARA*/PPARα, its contribution to metabolic remodeling is uncertain.

**Methods:**

Luciferase assays were performed to verify that TFEB regulates *PPARGC1A* expression. Cardiomyocyte-specific *Tfeb* knockout (cKO) and wildtype (WT) male mice were subjected to 27G transverse aortic constriction or sham surgery for 21 and 56 days, respectively, to induce LVH and HFrEF. Echocardiographic, morphological, and histological analyses were performed. Changes in markers of cardiac stress and remodeling, metabolic shift and oxidative phosphorylation were investigated by Western blot analyses, mass spectrometry, qRT-PCR, and citrate synthase and complex II activity measurements.

**Results:**

Luciferase assays revealed that TFEB increases *PPARGC1A*/PGC-1α expression, which was inhibited by class IIa histone deacetylases and derepressed by protein kinase D. At baseline, cKO mice exhibited a reduced cardiac function, elevated stress markers and a decrease in FAO and GO gene expression compared to WT mice. LVH resulted in increased cardiac remodeling and a decreased expression of FAO and GO genes, but a comparable decline in cardiac function in cKO compared to WT mice. In HFrEF, cKO mice showed an improved cardiac function, lower heart weights, smaller myocytes and a reduction in cardiac remodeling compared to WT mice. Proteomic analysis revealed a comparable decrease in FAO- and increase in GO-related proteins in both genotypes. A significant reduction in mitochondrial quality control genes and a decreased citrate synthase and complex II activities was observed in hearts of WT but not cKO HFrEF mice.

**Conclusions:**

TFEB affects the baseline expression of metabolic and mitochondrial quality control genes in the heart, but has only minor effects on the metabolic shift in LVH and HFrEF in mice. Deletion of TFEB plays a protective role in HFrEF but does not affect the course of LVH. Further studies are needed to elucidate if TFEB affects the metabolic flux in stressed cardiomyocytes.

## Introduction

Pathological left ventricular hypertrophy (LVH) and remodeling often cause heart failure (HF) with reduced ejection fraction (HFrEF), which increases morbidity and mortality of affected patients (1). Pathological LVH is an inappropriate response of the heart to various stress stimuli, such as pressure overload (PO) due to aortic valve stenosis or arterial hypertension (2) that is accompanied by cardiomyocyte hypertrophy, interstitial and perivascular fibrosis, and metabolic remodeling (3–6). At a molecular level, PO leads to an increase in cardiac stress markers, such as atrial natriuretic factor (ANF*/NPPA*), B-type natriuretic peptide (BNP/*NPPB*), and α-skeletal actin (ACTS/*ACTA1*) as well as a switch from the alpha myosin heavy chain (α-MyHC/*MYH6*) to the beta MyHC (β-MyHC/*MYH7*) isoform (3). Importantly, cardiac energy metabolism that is required for effective ATP production is greatly disturbed in LVH and HFrEF (7). ATP is essential to maintain contractile function, ion-homeostasis, and signal transduction in cardiomyocytes. To generate the high amounts of ATP the heart needs to enable its proper and continuous function as well as adaptations to changes in workload, the heart predominantly uses free fatty acids (FFA) and to a smaller extend glucose, lactate, ketone bodies and amino acids to generate ATP (7). These substrates are utilized in fatty acid β-oxidation (FAO) and glucose oxidation (GO) to produce acetyl coenzyme A (CoA) that is used by the tricarboxylic acid (TCA) cycle to produce NADH, which then undergoes oxidative phosphorylation (OXPHOS) in mitochondria. Specifically, complexes I–IV of the electron transport chain (ETC) transfer electrons from NADH to oxygen that causes a proton electrochemical gradient across the inner mitochondrial membrane, which is used by the ATP synthase to generate ATP. In LVH, a substrate shift from FAO to GO, known as metabolic remodeling (8, 9), leads to a less efficient ATP production that contributes to cardiomyocyte hypertrophy and cardiac dysfunction (10). The ATP content of the heart is relatively small and exhausts rapidly. Because cardiac function depends on efficient ATP production its impairment almost immediately causes a functional decline. The substrate shift in LVH is characterized by a decrease in FAO and a transient up-and subsequent downregulation of GO (7, 11–13) and increases the reliance of the heart to glucose (10). Because peroxisome proliferator–activated receptor α (PPARα, encoded by *PPARA*) and PPARα coactivator 1α (PGC-1α, encoded by *PPARGC1A*) regulate the expression of FAO-, GO- and OXPHOS-related genes, both transcriptional regulators are central to energy homeostasis and metabolic remodeling (14, 15). For example, the downregulation of PPARα in HFrEF causes a decrease in FAO (16). Likewise, cardiac ATP production of *Ppara* knockout mice depends on glucose, which is enabled by an increased glucose transporter type 4 (GLUT4) expression enhancing glucose uptake (17, 18). A decrease in PGC-1α was associated with a reduced ETC activity and an impaired OXPHOS-mediated ATP production. The expression of both, *PPARGC1A* (19) and *PPARA* (19, 20) as well as carnitine palmitoyltransferase I (*CPT-1*), the rate limiting enzyme in long-chain FAO, and GLUT4 is regulated by transcription factor EB (TFEB). TFEB is also involved in mitochondrial biogenesis and mitophagy indicating that it plays a role in cardiac energy homeostasis (20, 21). Previously we showed that cardiomyocyte-specific TFEB-overexpression by AAV2.9-mediated gene transfer caused HFrEF and excessive interstitial fibrosis when these mice were subjected to pressure-overload (22). However, the effects of TFEB overexpression on metabolic remodeling were not investigated. We also reported that TFEB binds to well conserved E-box elements of the human *TRIM63*-promoter that increases its expression and that this effect is inhibited by class IIa histone deacetylases (HDAC4, HDAC5, HDAC7). The protein kinase D (PKD)-family members PKD1, PKD2, and PKD3 attenuated this effect (23, 24). However, it has not been shown if the activity of TFEB towards the *PPARGC1A* promoter is also regulated by the PKD/HDAC axis. Additionally, we and others reported that the PKD- (25) and HDAC-families (26–29) are involved in pathological LVH. Specifically, cardiomyocyte-specific *Prkd1* knockout mice show less hypertrophy and myocardial remodeling in response to PO, β-adrenergic stimulation with isoproterenol, and Angiotensin II treatment (25). In contrast, cardiomyocyte-specific overexpression of PKD1 caused cardiac hypertrophy and heart failure. Also, deletion of *Hdac5* and *Hdac9* (28, 29) sensitizes the heart to stress signals. Because the PKD-family and class IIa HDACs regulate the activity of TFEB we hypothesized that the PKD/HDAC/TFEB-axis controls the expression of metabolic genes such as *PPARGC1A* and thus affects metabolic cardiac remodeling during stress. Therefore, we tested the hypothesis that TFEB in cardiomyocytes regulates PO-induced changes in proteins involved in metabolic remodeling and functional deterioration.

## Materials and methods

### Animal model

The *Landesamt für Landwirtschaft, Lebensmittelsicherheit und Fischerei* (LALLF, Mecklenburg-Vorpommern, Germany) approved the animal studies (permit number: 7221.3-1.1-014/18). The investigation conforms to the *Guide for the Care and Use of Laboratory Animals* published by the US National Institutes of Health (NIH Publication No. 85-23, revised 1985), as well as the current version of German Law on the Protection of Animals. Cardiomyocyte-specific *Tfeb* knockout mice (cKO, *Tfeb*^loxP/loxP; +/*α*MHC−CRE^) were generated by breeding *Tfeb*^loxP/loxP; +/+^ and αMHC-CRE transgenic mice (30), that conditionally express CRE recombinase under the control of a cardiomyocyte-specific αMHC promoter enabling cardiomyocyte-specific deletion of *Tfeb*. Genotyping was performed using primer pairs shown in Supplementary Table S1. *Tfeb*^loxP/loxP^ mice were kindly provided by Prof. A. Ballabio and Prof. C. Settembre (19). Mice from *Tfeb*^loxP/loxP; +/+^ and *Tfeb*^loxP/loxP; +/*α*MHC−CRE^ breeding’s were born in Mendelian distribution according to their genotype and no perinatal mortality of cKO mice was detected. During the observation period in the laboratory (approximately 42 weeks), WT and cKO mice did not show any differences in growth and weight or overall lifespan. No phenotypic differences were noticed between male and female mice. To induce PO, 8-week-old male mice were subjected to transverse aortic constriction (TAC), introducing a 27G stenosis as previously described (25, 31, 32). 21 days and 56 days of TAC were used to induce compensated LVH and HFrEF, respectively. The effectiveness of TAC was confirmed by pulsed-wave Doppler imaging from the left and the right carotid arteries [TAC vs. Sham mice; peak aortic valve (AV) velocity > 4,000 mm/s] as recently published (31, 32). Sham mice were treated identical for 21 days and 56 days, respectively, except for the ligation of the thoracic aorta. The number of animals was as follows: 21 days (TAC: cKO: *n* = 19 (2†,2*); WT: *n* = 19 (5†,1*); Sham: cKO: *n* = 10; WT: *n* = 10); 56 days (TAC: cKO: *n* = 17 (6†,5*); WT: *n* = 17 (7†,4*); Sham: cKO: *n* = 8; WT: *n* = 10) [† = died before the end of the experiment, * = A peak AV velocity (mm/s) of ≥4,000 was not achieved].

At the experimental endpoint, mice were anesthetized with 2% isoflurane for echocardiography and sacrificed thereafter during anesthesia by i.p. injection of a lethal dose of thiopental (200 mg/kg; Inresa Arzneimittel GmbH, Germany) and subsequent cervical dislocation. During necropsy, the integrity of the aortic banding was confirmed by inspection of the surgical constriction in TAC mice. Hearts, lungs, and livers were harvested and weighted. Body weight, organ weights, and tibial length were measured, and organ weights [mg] were normalized to tibia length [mm] and are shown as organ weight to tibial length ratios [mg/mm].

### Transthoracic echocardiography

Two-dimensional transthoracic echocardiography was performed as previously described (25, 31–35). Mice were anesthetized with 2% isoflurane and kept warm on a 37°C heated platform. Core body temperature, heart rate and rhythm were continuously monitored using a rectal probe and electrocardiography, respectively. For echocardiography a VisualSonics Vevo 2,100 High-Resolution Imaging System with a high-resolution (38 MHz) transducer was used. The examiner was blinded for genotypes and treatments. Following parameters were measured: thickness of left ventricular posterior wall (LVPWth; s, LVPWth; d), thickness of left ventricular anterior wall (LVAWth; s, LVAWth; d) and septum (IVSth; s, IVSth; d) at systole (s) and diastole (d), left ventricular end-diastolic (LVEDD) and end-systolic (LVESD) dimensions. Calculated parameters are summarized in Table 1.

**Table 1:** Calculated echocardiographic parameters.

Label	Description	Units	Formula
LVESV	Left ventricular end-systolic volume	µl	[7.0/(2.4 + LVESD)]*LVESD^3^
LVEDV	Left ventricular end-diastolic volume	µl	[7.0/(2.4 + LVEDD)]*LVEDD^3^
LVSV	Left ventricular stroke volume	µl	LVEDV—LVESV
LVEF	Left ventricular ejection fraction	%	100*[(LVEDV—LVESV)/LVEDV]
LVFS	Left ventricular fractional shortening	%	100*[(LVEDD—LVESD)/LVEDD]
CO	Cardiac output	µl/min	HR*LVSV
LVM	Left ventricular mass	mg	1.053*[(LVEDD + LVPWd + IVSd)^3^—LVEDD^3^]

### Histological analyses

Hearts were formalin-fixed and embedded in paraffin. A rotary microtome (Microm HM 340 E, Thermo Fischer Scientific Inc., MA, USA) was used to prepare 6 µm thin myocardial cross-sections, which were stained with Hematoxylin and Eosin (H&E), Picrosirius Red (PSR), and Periodic acid–Schiff (PAS) (25, 33, 35). H&E: Histological sections were deparaffinized in xylol (Cat. no.: 9713.1, Carl Roth GmbH, Karlsruhe, Germany) and rehydrated in a descending ethanol dilution series (i.e., 100%, 96%, 80%, 70%). They were then stained with hematoxylin (Cat. no.: 3816.2, Carl Roth GmbH) and eosin (Cat. no.: 7089.1, Carl Roth GmbH), dehydrated with 95% and 100% ethanol (Carl Roth GmbH) followed by xylene treatment, and mounted with coverslips. PSR: histological cross-sections were fixed in xylol (Carl Roth GmbH) and hydrated with a descending ethanol dilution series (i.e., 100%, 96%, 80%, 70%, 50%, 30%). Sections were then stained in Picrosirius Red F3BA (Cat. no.: NC9039835, Polysciences Inc., PA, USA), dehydrated with xylol and mounted with VectaMount® permanent mounting medium (Cat. no.: 101098-068, Vector Laboratories Inc., Newark, State, USA). PAS: To stain the glycogen content, sections were stained with periodic acid (Cat. no.: 3257.1, Carl Roth GmbH), washed, and then stained with Schiff’s reagent (Cat. no.: X900.1, Carl Roth GmbH). Nuclei were stained with hematoxylin (Carl Roth GmbH). Wheat Germ Agglutinin (WGA) staining: sections were deparaffinized in NeoClear (Cat. no.: A538.1, Carl Roth GmbH) and rehydrated in a descending ethanol dilution series (i.e., 100%, 96%, 80%, 70%), washed with 1× phosphate buffered saline (PBS) and blocked with 5% donkey serum for 1 h. Sections were then stained with WGA-FITC (Cat. no.: FL-1021, Vector Laboratories Inc.) for 2 h at room temperature (RT). Sections were covered with Ibidi mounting medium (Cat. no.: 50001, ibidi GmbH, Gräfelfing, Germany) and stored at −20°C until analyses. Images were acquired using a Keyence BioRevo BZ-9000 imaging system (Keyence Deutschland GmbH, Germany). Myocardial fibrosis, glycogen content, and myocyte cross-sectional areas (MCSA) were quantified using ImageJ software 1.52 (http://rsb.info.nih.gov/ij).

### RNA isolation, cDNA synthesis, and quantitative real-time PCR

Total RNA was isolated from the interventricular septum and left ventricle using TRIzol® reagent (Cat. no.: 15596026, Invitrogen™, Life Technologies Corp., CA, USA), and cDNA was synthesized using the SuperScript® first-strand synthesis kit (Cat. no.: 10684803, Invitrogen) as published previously (24, 36, 37). Quantitative real-time polymerase chain reaction (qRT-PCR) was performed using power SYBR® Green PCR master mix (Cat. no.: A25778, Applied Biosystems, Thermo Fischer Scientific Inc.) and self-designed primers (for primer sequences see Supplementary Table S2) on a QuantStudio 3 (Applied Biosystems, MA, USA) using the standard curve method as described previously (33, 36, 38, 39). Gene expression was normalized to the expression levels of the stably expressed reference gene *glyceraldehyde-3-phosphate dehydrogenase* (*Gapdh*).

### Protein extraction and western blot analyses

BeadBlaster™ 24 Microtube Homogenizer (Benchmark Scientific, Sayreville, NJ, USA) was used to homogenize heart tissue in Micro Packaging Vials with 2.8 mm Precellys ceramic beads (PEQLAB Biotechnology GmbH, Germany) in UT-buffer (8 M urea, 2 M thiourea). Lysates were cleared by centrifugation at 17,000 g for 30 min at 4°C and protein content was quantified using Bradford assay (BioRad, Munich, Germany). After adding Laemmli buffer (final concentration: 300 mM Tris-HCl, pH 6.8; 6% (w/v) SDS; 0.05% (w/v) bromophenol blue, 10% (v/v) glycerol and 15% (v/v) β-mercaptoethanol), samples were heated for 5 min at 95°C and afterwards proteins were resolved by SDS-PAGE and transferred onto nitrocellulose membranes (Cat. no.: GE10600001, GE Healthcare, Munich, Germany). Membranes were blocked with 5% bovine serum albumin (BSA) in TBS-T (20 mM Tris, 150 mM NaCl, 0.1% Tween 20; pH 7.6) for 1 h. Following primary antibodies were used: affinity-purified rabbit anti-TFEB antibody A303-673A (RRID: AB_11204751, 1:1000, Bethyl Laboratories, Hamburg, Germany), monoclonal anti-β/slow myosin heavy chain (β-MyHC) (RRID: AB_297734, clone: NOQ7.5.4D, mouse, 1:1,000, Merck KGaA, Munich, Germany). Anti-glyceraldehyde-3-phosphate dehydrogenase (RRID: AB_2756824, GAPDH, D4C6R, mouse, 1:10,000, Cell Signaling, MA, USA) was used as loading control. Horseradish peroxidase (HRP)-linked IgG goat anti-mouse (RRID: AB_330924) and goat anti-rabbit (RRID: AB_2099233, both 1:10,000, Cell Signaling) were used as secondary antibodies. Proteins were visualized with a chemiluminescence system (ChemiDoc MP Imaging System, Bio-Rad).

### Spectrophotometric measurement of complex II and citrate synthase activity

Enzyme activity for (ETC) complex (C) II was quantified according to a previously published protocol (40). Briefly, measurements were performed at RT using an UV/Visible spectrophotometer (UV-1600PC, VWR International Europe BVBA, Leuven, Belgium), and in technical triplicates. Heart tissues were homogenized in ice-cold homogenization buffer (20 mM Tris, 40 mM KCl, 250 mM sucrose, 2 mM EGTA, pH 7.4) using a tissue grinder. For CII activity 5 µg of protein homogenate was added to a 1 ml cuvette containing potassium phosphate buffer (0.5 M, pH 7.5), fatty acid-free BSA (0.75 mM), KCN (10 mM), succinate (20 mM) and 2,6-Dichlorophenolindophenol (DCPIP, 80 µM). Samples were incubated for 10 min at 37°C. After baseline measurements (OD 600 nm) the reaction was primed by adding decylubiquinone (50 µM) and the changes in OD 600 nm (*Δ*OD) were recorded for 3 min. The malonate-insensitive CII activity was determined simultaneously by adding the CII inhibitor malonate (1 M) into the reaction mixture. For the citrate synthase activity (CS) activity assay 5 µg of protein homogenate was added to a 1 ml cuvette containing 100 mM Tris, pH 8.0, 0.2% (vol/vol) Triton X-100, 5,5’-Dithiobis (2-nitrobenzoic acid) DTNB (100 µM), and Acetyl-CoA (300 µM). The baseline OD was read at a wavelength of 412 nm for 3 min. The reaction was started by adding of oxaloacetic acid (10 mM), and the increase in absorbance at OD 412 nm was monitored for 3 min. Enzyme activities were calculated using extinction coefficients (*ε*; mmol^−1^ cm^−1^) (CII *ε* = 19.1, CS *ε* = 13.6). In the calculation's CS and CII activities measured in proteins from hearts of WT Sham mice, respectively, were set to 100% and all other activities were related to that.

### Cell culture, cDNA expression plasmids, transfection, and luciferase reporter assays

COS-7 cells (RRID: CVCL_0224) were cultured in Dulbecco’s modified eagle medium (DMEM; 4.5 g/l glucose, L-glutamine, 10% fetal bovine serum, and penicillin/streptomycin) (23, 24). Cells were transfected with cDNA expression plasmids, vector control, and Hs_*PPARGC1A*_GLuc-ON (GeneCopoeia Inc., MD, USA) reporter construct, as indicated, using FuGENE®6 (Promega, Madison, WI, USA) transfection reagent according to the manufacturer's protocol. To control transfection efficacy 20 ng of pCMV lacZ (RRID: Addgene_31124, CLONTECH Laboratories GmbH, Heidelberg, Germany) was co-transfected in each sample. Cell pellets were lysed in 100 µl cell lysis buffer (Cat. no.: 16161, Merck KGaA). 20 µl of cell culture supernatant was used for quantification of luciferase and β-galactosidase activity in an Infinite M200 Pro spectrophotometer (Tecan, Maennedorf, Switzerland). The Gaussia luciferase glow assay kit (Cat. no.: 16161, Merck KGaA) was used to quantify the expression of the reporter gene constructs. Luciferase activity was normalized to fluorescence measured with the FluoReporter® lacZ/galactosidase quantification kit (Cat. no.: F2905, Invitrogen). The plasmids used for cDNA expression (pcDNA3.1-TFEB-N-FLAG, pcDNA3.1-TFE3-N-FLAG, pcDNA3.1-PKD1-CA-N-MYC, pcDNA3.1-PKD2-CA-N-MYC, pcDNA3.1-PKD3-CA-N-MYC, pcDNA3.1-HDAC4-MYC, pcDNA3.1-HDAC5-MYC, pcDNA3.1-HDAC7-MYC, pcDNA3.1-C-MYC, pCMV lacZ) were recently published (23–25, 41, 42).

### Mass spectrometric analysis

For preparation of each sample, five µg of protein extracted from the cardiac apex was utilized (6 bioreplicates per condition, i.e., WT Sham, WT TAC, cKO Sham, cKO TAC). For nucleic acid degradation we added 0.625 U benzonase before reduction (2.5 mM DTT ultrapure, Cat. no.: 11568736, Invitrogen, for 15 min at 37°C) and alkylation (10 mM iodoacetamide, Cat. no.: I1149, Merck KGaA, for 30 min at 37°C). Proteolysis with LysC (Cat. no.: VA1170, 1:100 for 3 h at 37°C) followed by tryptic digestion over night at 37°C (Cat. no.: VA9000, both from Promega) and purification of peptides was performed with a bead-based protocol (43). Desalted peptides were analyzed by LC-ESI tandem mass spectrometry on an Exploris 480 mass spectrometer (Thermo Electron, Bremen, Germany) in data independent acquisition mode (Supplementary Tables S3A,B). Peptide and protein identification were carried out using a directDIA algorithm with an Uniprot database limited to murine entries (02/2021) implemented in Spectronaut (v. 14.10.201222.47784, Biognosys, Schlieren, Switzerland). We extracted quantitative data by Spectronaut™ based on MS2 peak areas. Missing values were parsed using an indexed retention time (iRT) profiling strategy (minimum Q-value row selection = 0.001). Only non-identified precursors were parsed with a Q-value > 0.001. Ion values were parsed when at least 25% of the samples contained high quality measured values. Peptides were assigned to protein groups and protein inference was resolved by the automatic workflow implemented in Spectronaut™. We considered only proteins with at least two quantified peptides for further analyses. Protein intensities were calculated as MaxLFQ values. Data have been median normalized on ion level before statistical analysis was carried out on peptide level after exclusion of peptides with oxidized methionine using the algorithm ROPECA (44). Binary differences were identified by application of a reproducibility-optimized test statistic (using the ROTS package). Multiple test correction was performed according to Benjamini-Hochberg. Variance within the data set was visualized by principal component analyses and differences in the protein pattern by Volcano plots.

### Ingenuity pathway analysis of proteomics data

For functional classification and analysis of proteins displaying significantly altered levels Ingenuity® Pathway Analysis (IPA®, QIAGEN Redwood City, www.qiagen.com/ingenuity) was used. An adjusted *p*-value (*q*-value) of <0.05 was considered statistically significant. The IPA upstream regulator analytics identifies upstream transcription regulators based on the observed changes in the protein abundance in the dataset. The results of the calculation are shown as activation z-score, an z-score >2 represents a significant activation and a z-score <−2 represents a significant inhibition of a protein or pathway.

### Statistical tests

In comparisons of two groups of histological staining's from mouse samples were analyzed by an unpaired *t*-test. Differences of *p < *0.05 were considered statistically significant. In comparisons of two groups, mRNA and Western blot data were analyzed by multiple *t*-tests with FDR adjustment [two-stage-step-up method of Benjamini, Krieger and Yekutieli (45)]. In comparisons of four groups, data were analyzed by One-way ANOVA with Tukey's *post-hoc* test. For echocardiography of 21 days of TAC, all time points were analyzed by Two-way ANOVA with Tukey's *post-hoc* test. Echocardiography of baseline and 56 days of TAC were analyzed using linear mixed effects models. We included measurement time point (weeks), genotype (cKO vs. WT), and surgery type (TAC vs. Sham) as fixed effects and animal-ID as random factor. We included all possible 2-way and 3-way interactions terms in the initial full model and used a backward elimination algorithm to get rid of unnecessary interaction terms. All statistical were performed using R version 4.2.2 with packages -lmerTest-, -car-, and -emmeans-. *Post-hoc* comparisons were calculated based on the final model per echocardiography parameter using consecutive pairwise comparison between the different time points in each treatment combination.

Pairwise comparisons between the resulting four treatment groups (WT Sham, WT TAC, cKO Sham, cKO TAC) were done at each time point. All values are shown as mean ± 95% CI. The significance level was set to α = 0.05. Model assumptions were ensured by visually checking QQ-plots for normality of residuals and residual-vs.-fitted plots for homoscedasticity. Differences of *q < *0.05 were considered statistically significant. Two-tailed Pearson correlation analyses among variables of log_2_ ratios WT_TAC/WT_Sham and cKO_TAC/cKO_Sham or cKO_TAC/WT_TAC or cKO_Sham/WT_Sham were performed. Data are presented as box-and-whisker plots showing all data points and boxes showing median and interquartile range (IQR) and whiskers indicating minimum and maximum values. Plots and statistics were performed using GraphPad Prism® 8.3.0 program (GraphPad Software, Boston, MA, USA; version 8.3.0).

## Results

### TFEB-mediated *PPARGC1A* expression is controlled by class IIa histone deacetylases and the protein kinase D family

The metabolic shift from FAO to GO in LVH is controlled by PGC-1α/*PPARGC1A*, a master regulator of mitochondrial biogenesis and FAO (46, 47). TFEB was shown to increase the expression of *PPARGC1A* and *PPARA* (20, 48). Previously, we and others reported that the PKD family (25) and class IIa HDACs (26–29) are involved in pathological LVH and that the PKD/HDAC axis regulates the activity of TFEB towards the human *Hs_TRIM63*/MuRF1 promoter (23, 24). If TFEB-mediated *PPARGC1A* expression is controlled by class IIa HDACs and PKD was not known.

**Figure 1:**
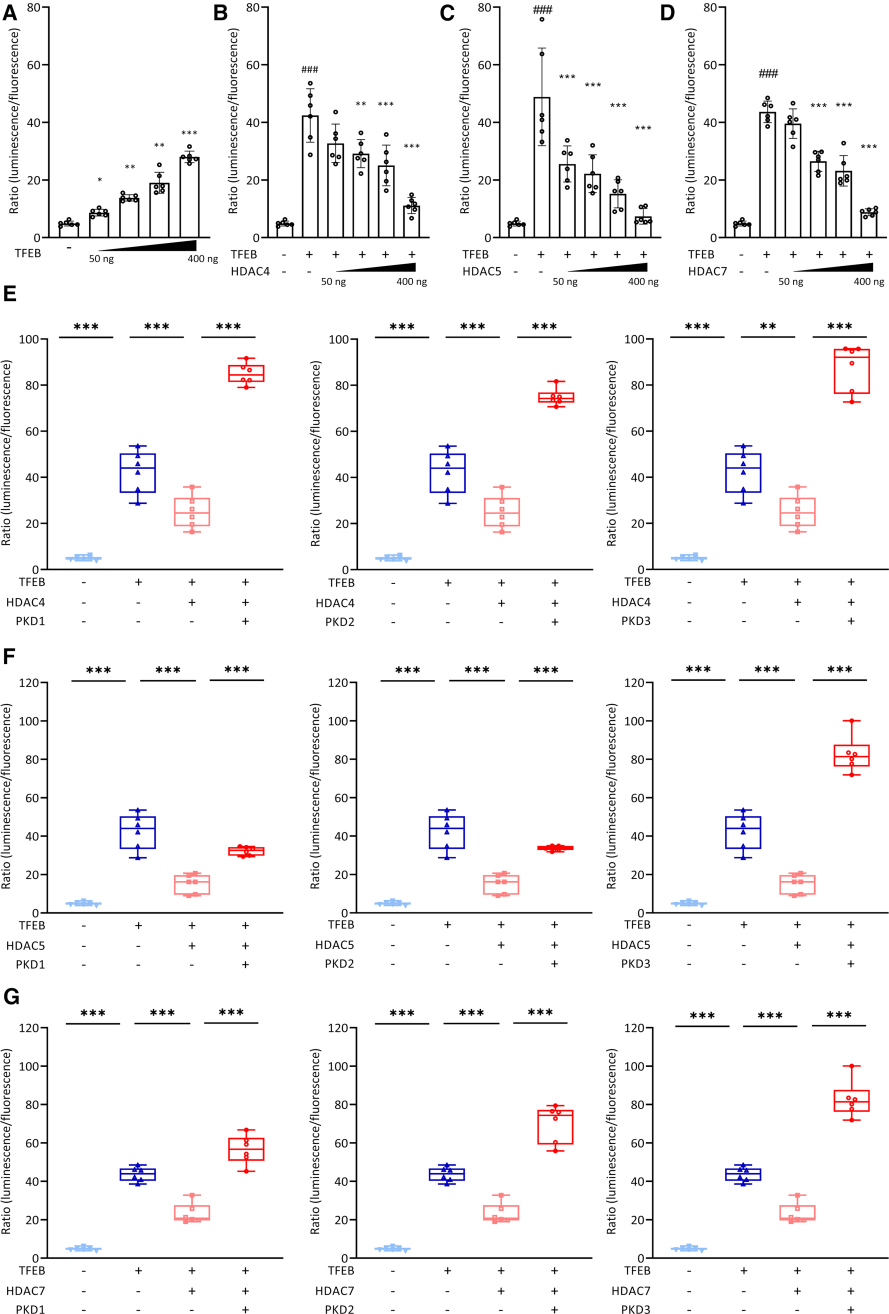
TFEB-induced *PPARGC1A* expression is regulated by class IIa HDACs and PKD. (**A**) Luciferase assays performed with cell extracts of COS-7 cells transfected with Hs_*Ppargc1a*-Luc and increasing amounts of FLAG-TFEB (TFEB; 50, 100, 200, and 400 ng) as indicated or control (-) plasmid. Luciferase activity was normalized to expression of CMV-LacZ and expressed as fold increase. Data are represented as mean ± SD. Two-tailed *t*-test; *p < 0.05, **p < 0.01, ***p < 0.001 vs. transfection with TFEB only. (**B–D**) Luciferase assays performed with cell extracts of COS-7 cells transfected with Hs_*Ppargc1a*-Luc, FLAG-TFEB (TFEB; 400 ng) and increasing amounts (50, 100, 200, and 400 ng) of MYC-HDAC4 (HDAC4), MYC-HDAC5 (HDAC5), or MYC-HDAC7 (HDAC7) as indicated or control (-) plasmid. Luciferase activity was normalized to expression of CMV-LacZ and expressed as fold increase. Data are represented as mean ± SD. One-way ANOVA *q* < 0.0001 for (**B**); ###*q *< 0.001 vs. control transfection with pcDNA3.1; *q < 0.05; **q < 0.01; ***q < 0.001 vs. transfection with TFEB only. (**E–G**), COS-7 cells were transfected with expression plasmids encoding FLAG-TFEB, (**E**) HDAC4-MYC, (**F**) HDAC5-MYC, or (**G**) HDAC7-MYC, or constitutively active (ca) PKD1 (left panel), caPKD2 (middle panel), and caPKD3 (right panel) proteins, as indicated, together with the Hs_*Ppargc1a*-Luc reporter construct. Values were normalized to expression of CMV-LacZ and calculated as the fold increase in luciferase/CMV-LacZ ratio compared with the reporter alone. Data are represented as mean ± SD. One-way ANOVA *q *< 0.0001 for (**E–G**); *q < 0.05; **q < 0.01; ***q < 0.001. *n* = 5.

Using luciferase assays, we confirmed that TFEB increases the expression of *PPARGC1A* in a dose dependent manner (Figure 1A). Co-transfection of HDAC4 (Figure 1B), HDAC5 (Figure 1C), and HDAC7 (Figure 1D) dose-dependently inhibited TFEB-induced *PPARGC1A*-expression. Previously, we reported that PKD1 and PKD2 associate with, phosphorylate, and facilitate 14-3-3-mediated nuclear export of HDAC4, HDAC5, and HDAC7. This relieved the inhibition of TFEB towards the human *Hs_TRIM63*/MuRF1 promoter (23, 24). In the luciferase assay, we observed that the repressive effects of HDAC4 (Figure 1E), HDAC5 (Figure 1F), and HDAC7 (Figure 1G) on TFEB-induced *PPARGC1A*-activity was reversed by PKD1 (Figures 1E–G, left panels), PKD2 (Figures 1E–G, middle panels), and PKD3 (Figures 1E–G, right panels), respectively. These data indicate that the PKD family converges on HDAC4, HDAC5, and HDAC7 to control TFEB-induced *PPARGC1A* expression. Because TFEB and TFE3 have partially redundant functions (23, 49, 50), we investigated if TFE3 also increases the expression of *PPARGC1A*. Indeed, TFE3 increased the expression of *PPARGC1A* in a dose dependent manner (Supplementary Figure S1A), which was reduced by HDAC4 (Supplementary Figure S1B), HDAC5 (Supplementary Figure S1C), and HDAC7 (Supplementary Figure S1D). These repressive effects were reversed by co-transfection of PKD1 (Supplementary Figures S1E–G, left panels), PKD2 (Supplementary Figures S1E–G, middle panels), and PKD3 (Supplementary Figures S1E–G, right panels). Our data indicate that both TFEB and TFE3 increased the expression of *PPARGC1A* and that both transcription factors are regulated by class IIa HDACs and the PKD family.

### Deletion of *Tfeb* is accompanied by increased cardiac stress and remodeling as well as a reduced cardiac function

To test the hypothesis that TFEB is involved in PO-induced regulation of metabolic genes in cardiomyocytes, we generated cardiomyocyte-specific *Tfeb* knockout mice (*Tfeb*^loxP/loxP; *α*MHC−CRE^; cKO) and used wildtype littermates (*Tfeb*^loxP/loxP^; WT) as controls. Baseline analyses confirmed a decrease in TFEB mRNA expression (to 24%; Supplementary Figure S2A) and protein content (to 25%; Supplementary Figure S2B) in cKO compared to WT hearts. A compensatory increase in *Tfe3* and *Mitf* expression was not observed in cKO hearts (Supplementary Figure S2A). No differences in heart (HW/TL), lung (Lu/TL) and liver (Li/TL) weight-to-tibia length ratios were found between cKO and WT mice (Supplementary Figure S2C). Transthoracic echocardiography revealed a decrease in LV ejection fraction (LVEF) and fractional shortening (FS) in cKO mice (Supplementary Figure S2D). Analysis of hematoxylin-eosin (H&E) stained histological cross-sections showed no morphological differences between both genotypes (Supplementary Figure S2E). Quantification of myocyte cross-sectional areas (MCSA) on WGA-stained histological sections (Supplementary Figure S2F) revealed no differences in cardiomyocyte size between cKO and WT mice (Supplementary Figure S2G). The expression of the cardiac stress markers *Nppa*, *Nppb* and *Myh7* was increased and *Myh6* was decreased in hearts of cKO compared to WT mice (Supplementary Figure S2H). Western blot analyses showed a significant increase of β-MyHC in cKO compared to WT hearts (Supplementary Figure S2I). Picrosirius Red (PSR) staining of histological cross-sections showed a trend towards interstitial fibrosis (*p *= 0.054) (Supplementary Figure S2J), which was paralleled by an increased expression of the extracellular matrix proteins *Collagen alpha-1(I) chain* (*Col1a1*) and *Fibronectin* (*Fn*) whereas *Collagen alpha-1(III) chain* (*Col3a1*) and *connective tissue growth factor* (*Ctgf*) remained unchanged in cKO compared to WT mice (Supplementary Figure S2K). These data show that cardiomyocyte-specific TFEB-deletion is accompanied by a reduced cardiac function as well as an increase in cardiac stress and fibrosis markers.

### *Tfeb* cKO leads to a dysregulation of genes involved in energy homeostasis

The expression of the known TFEB target genes *Ppargc1a*, *Ppara*, and *Cpt1b* was significantly reduced in hearts of cKO compared to WT mice (Supplementary Figure S3A) indicating that TFEB is involved in their baseline expression. PGC-1α is a nodal factor in mitochondrial biogenesis and quality control (51). Once activated, PGC-1α increases nuclear respiratory factor-1 (*Nrf1*) and its canonical downstream target mitochondrial transcription factor A (*Tfam*) to stimulate mitochondrial biogenesis and mitochondrial energy metabolism (51). To investigate if the reduction in *Ppargc1a* expression has an effect on its downstream targets, we quantitated the *Nrf1* and *Tfam* expression and found a trend towards a reduction in hearts of cKO mice (Supplementary Figure S3A). We also analyzed the expression of the orphan nuclear receptors estrogen related receptors α, β, and γ (*Esrra*, *Esrrb*, *Esrrg*) that cooperate with PGC-1α to increase the expression of FAO and OXPHOS genes in the heart (52–54). Further, *Esrra* is also regulated by TFEB (55). We observed a significant reduction of *Esrra* expression in hearts of cKO compared to WT mice, whereas *Esrrb* and *Esrrg* remained unchanged (Supplementary Figure S3B). Because PGC-1α regulates mitochondrial biogenesis we quantified the expression of ETC related genes (Supplementary Table S4) and found transcripts of *Sdha* and *Sdhb* (CII), *Uqcrc2* (CIII) and *Atp5a1* (CV) to be reduced in hearts of cKO mice (Supplementary Figure S3C). However, the expression of fission 1 (*Fis1*), dynamin-related protein 1 (*Drp1*), and mitofusin 1 (*Mfn1*) and *Mfn2* that are also regulated by PGC-1α and that regulate mitochondrial fission and fusion (47) remained unchanged between hearts of cKO and WT mice (Supplementary Figure S3D). In terms of glucose metabolism, we found a reduction in *Slc2a4* (encoding GLUT4) and *hexokinase-2* (*Hk2*) expression that mediates glucose degradation whereas *glycogen synthase 1* (*Gys1)* that mediates glycogen synthesis, and the glucose transporter *Slc2a1* (GLUT1) remained unchanged in cKO compared to WT mice (Supplementary Figure S3A). PAS staining indicated that the glycogen content was reduced in hearts of cKO compared to WT mice (Supplementary Figure S3E). The changes in gene expression between cKO and WT hearts are illustrated in Supplementary Figure S3D. Overall, our data indicate that TFEB is involved in baseline expression of metabolic genes in cardiomyocytes.

### Cardiomyocyte-specific deletion of TFEB augments cardiac stress response but has no effect on cardiac function during PO-induced LVH

**Figure 2:**
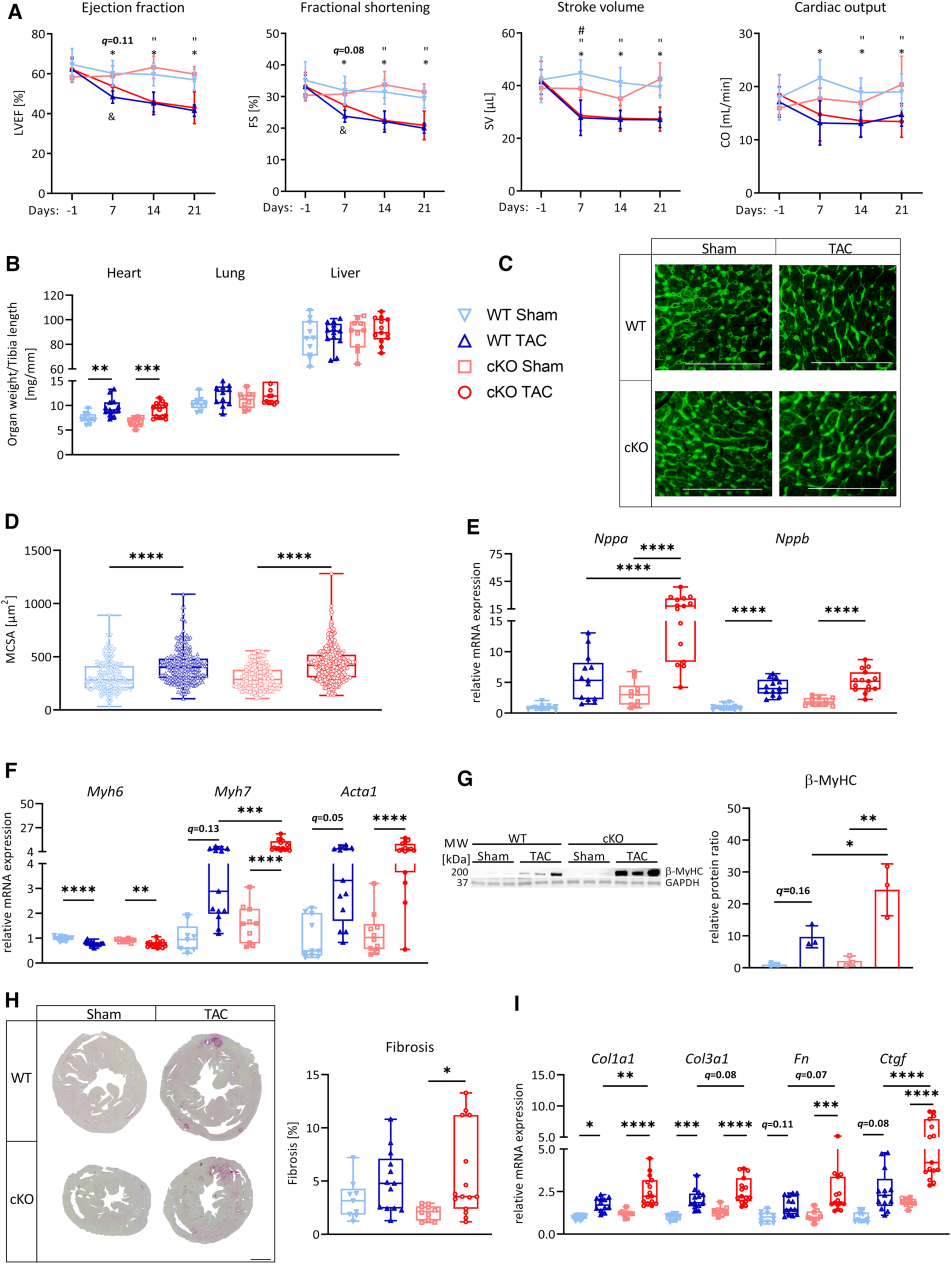
*Tfeb* cKO under pressure overload-induced LVH does not alter cardiac function. (**A**) Kinetics of left ventricular ejection fraction (LVEF), fractional shortening (FS), stroke volume (SV) and cardiac output (CO) as determined by echocardiography at indicated time points after Sham and TAC surgery, respectively, of WT and cKO mice (* = WT_TAC/WT_Sham, “ = cKO_TAC/cKO_Sham, & = cKO_TAC/WT_TAC). (**B**) Heart, lung and liver weights of WT and cKO mice after 21 days of Sham or TAC surgery, normalized to tibia length. (**C–D**) Wheat Germ Agglutinin (WGA) stained histological cross-sections of hearts from WT and cKO mice after 21 days of Sham or TAC surgery. (**D**) Myocyte cross-sectional area (MCSA) measured from on WGA stained sections with Image J. Scale bar, 100 µm. Quantitative real-time polymerase chain reaction (qRT-PCR) analysis of *Nppa* and *Nppb* (**E**) and *Myh6*, *Myh7* and *Acta1* (**F**) expression from WT and cKO mice after 21 days of Sham or TAC surgery as indicated. mRNA expression was normalized to *Gapdh*. (**G**) Western blot analysis with anti-β-MyHC and anti-GAPDH antibodies. GAPDH was used as loading control. Bar graph showing the ratio of the relative densities of β-MyHC and GAPDH protein contents. (**H**) Representative images of Picrosirius Red stained (PSR, left) heart cross-sections of WT and cKO mice after 21 days of Sham or TAC surgery; scale bar, 1 mm. Fibrotic area (right) was measured with Image J. (**I**) qRT-PCR analysis of *Col1a1*, *Col1a3*, *Fn*, and *Ctgf* from WT and cKO mice after 21 days of Sham or TAC surgery as indicated. mRNA expression was normalized to *Gapdh*. Data are presented as mean ± SD (WT Sham: *n* = 10, WT TAC: *n* = 13, cKO Sham: *n* = 10, cKO TAC: *n* = 15; ^*,”,&^*q *< 0.05, **q < 0.01, ***q < 0.001, ****q < 0.0001).

Because metabolic remodeling genes were decreased and cardiac stress markers were increased in hearts of cKO mice, we reasoned that cKO mice would be more susceptible for PO-induced myocardial stress. To test this hypothesis, we subjected male cKO mice (Sham: *n* = 10, TAC: *n* = 15) and WT littermate controls (Sham: *n* = 10, TAC: *n* = 13) to 27G TAC- or Sham-surgery for 21 days to induce LVH. PO did not affect the expression of *Tfeb*, *Tfe3,* or *Mitf* (Supplementary Figure S4A) in WT or cKO mice. Weekly performed transthoracic echocardiography showed a continuous increase in LV mass (Supplementary Figure S4B) and a decrease in LVEF, FS, SV, and CO (Figure 2A; Supplementary Table S5) in WT and cKO TAC mice compared to their respective Sham group without differences between the genotypes. Following 21 days of TAC an increase in HW/TL ratio was observed in both WT (+29% vs. WT Sham) and cKO (+36% vs. cKO Sham) mice, whereas Lu/TL and LiW/TL ratios remained unchanged (Figure 2B). Analyses of H&E- (Supplementary Figure S4C) and WGA-stained (Figures 2C,D) histological cross-sections showed enlarged MCSA (Supplementary Figures S4D–G) in TAC compared to Sham mice without differences between both genotypes. *Nppa* and *Nppb* expression was increased in cKO TAC, whereas only *Nppb* expression was elevated in WT TAC mice compared to their Sham groups. *Nppa* expression was significantly higher in cKO compared to WT TAC hearts (Figure 2E). The expression of *Myh6* was decreased and *Acta1* (WT: *q = *0.054, cKO: *q < *0.0001) was increased in WT and cKO TAC mice (Figure 2F). *Myh7* mRNA expression (WT: *q = *0.13, cKO: *q < *0.0001) and β-MyHC protein contents (WT: *q = *0.16, cKO: *q < *0.01) were increased in response to TAC, which was only significant in cKO hearts. The increase in *Myh7* mRNA and β-MyHC protein was significantly higher in cKO TAC compared to WT TAC mice (Figure 2G). PSR staining revealed an increased interstitial fibrosis in hearts of cKO but not WT TAC mice (Figure 2H). The expression of *Col1a1* and *Col3a1* was increased and the expression of *Fn* (*q = *0.11) and *Ctgf* (*q = *0.08) showed a trend towards an increase in WT TAC compared to WT Sham mice. The expression of *Col1a1*, *Col3a1*, *Fn*, and *Ctgf* was significantly higher in cKO TAC than in cKO Sham mice. *Col1a1* and *Ctgf* expression was significantly higher in cKO TAC compared to WT TAC mice and *Col3a1* (*q = *0.08) and *Fn* (*q = *0.07) showed a trend towards a higher expression in cKO TAC mice (Figure 2I). In summary, these data reveal that cardiac stress response and remodeling is more pronounced in cKO compared to WT hearts in response to PO-induced LVH, which however did not affect cardiac function.

### Absence of TFEB in cardiomyocytes has minor effects on metabolic remodeling genes in LVH

**Figure 3:**
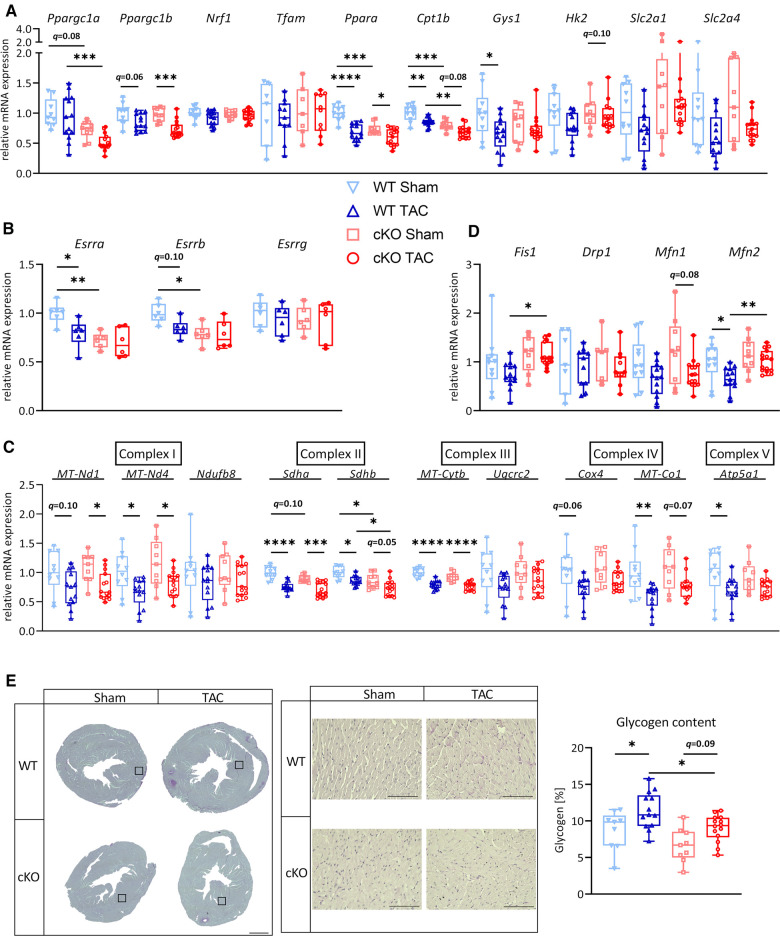
Deletion of TFEB has minor effects on energy homeostasis in LVH. Quantitative real-time polymerase chain reaction (qRT-PCR) analysis of indicated metabolic genes (**A–B**) and genes involved in OXPHOS (**C**) and mitochondrial fission and fusion (**D**) from WT and cKO mice after 21 days of Sham or TAC surgery as indicated. mRNA expression was normalized to *Gapdh*. (**E**) Representative images of PAS stained heart cross-sections of WT and cKO mice after 21 days of Sham or TAC surgery; left: overview, scale bare 1 mm; middle: higher magnification (corresponding to inset from left), scale bar 100 µm, right: quantification of glycogen content with Image J. Data are presented as mean ± SD (21 days, WT Sham: *n* = 10, WT TAC: *n* = 13, cKO Sham: *n* = 10, cKO TAC: *n* = 15; *q < 0.05, **q < 0.01, ***q < 0.001, ****q < 0.0001).

After 21 days of TAC, *Ppara* (WT: *q *< 0.0001, cKO: *q *< 0.05), *Cpt1b* (WT: *q *< 0.01; cKO: *q *= 0.078) and *Ppargc1b* (PGC**-**1β, WT: *q *= 0.055, cKO: *q *< 0.001) were downregulated or showed a trend towards downregulation in WT TAC and cKO TAC mice compared to their respective Sham group (Figure 3A). The expression of *Ppargc1a* and *Cpt1b* was significantly lower in cKO compared to WT TAC mice (Figure 3A). We found a significantly lower *Esrra* expression in cKO Sham compared to WT Sham and mice. TAC caused a significant reduction in *Esrra* expression in WT TAC compared to WT Sham hearts but did not affect *Esrra* expression in cKO TAC compared to cKO Sham hearts (Figure 3B). The expression of most ETC genes, such as *MT-Nd1* (WT: *q *= 0.10, cKO: *q *< 0.05), *MT-Nd4* (WT: *q *< 0.05, cKO: *q *< 0.05), *Sdha* (WT: *q *< 0.0001, cKO: *q *< 0.001), *Sdhb* (WT: *q *< 0.05, cKO: *q* = 0.05), *MT-Cytb* (WT: *q *< 0.0001, cKO: *q *< 0.0001), *Cox4* (WT: *q *= 0.06, cKO: n.s.), *MT-Co1* (WT: *q *< 0.01, cKO: *q *= 0.07) and *Atp5a1* (WT: *q *< 0.05, cKO: n.s.) was reduced in WT TAC and cKO TAC mice compared to their Sham group (Figure 3C). However, only the expression of *Sdhb* was significantly lower in cKO TAC compared to WT TAC mice (Figure 3C). The expression of *Fis1* and *Mfn2* was significantly higher in cKO TAC compared to WT TAC mice (Figure 3D). *Gys1* was decreased in WT TAC compared to WT Sham and *Hk2*, *Slc2a1*, and *Slc2a4* remained unchanged in all groups (Figure 3A). PAS staining revealed an increased glycogen content in response to TAC in both genotypes (WT: *q < *0.05, cKO: *q *= 0.09) but the glycogen content was lower in cKO TAC compared to WT TAC hearts (Figure 3E). These data are suggestive for an increased glycogen consumption in cKO compared to WT hearts in response to PO-induced LVH. Our results indicate that a metabolic gene switch occurs in the heart during LVH and that TFEB contributes to the expression of genes that are involved in myocardial energy homeostasis.

### Deletion of TFEB has beneficial effects in PO-induced HFrEF

**Figure 4:**
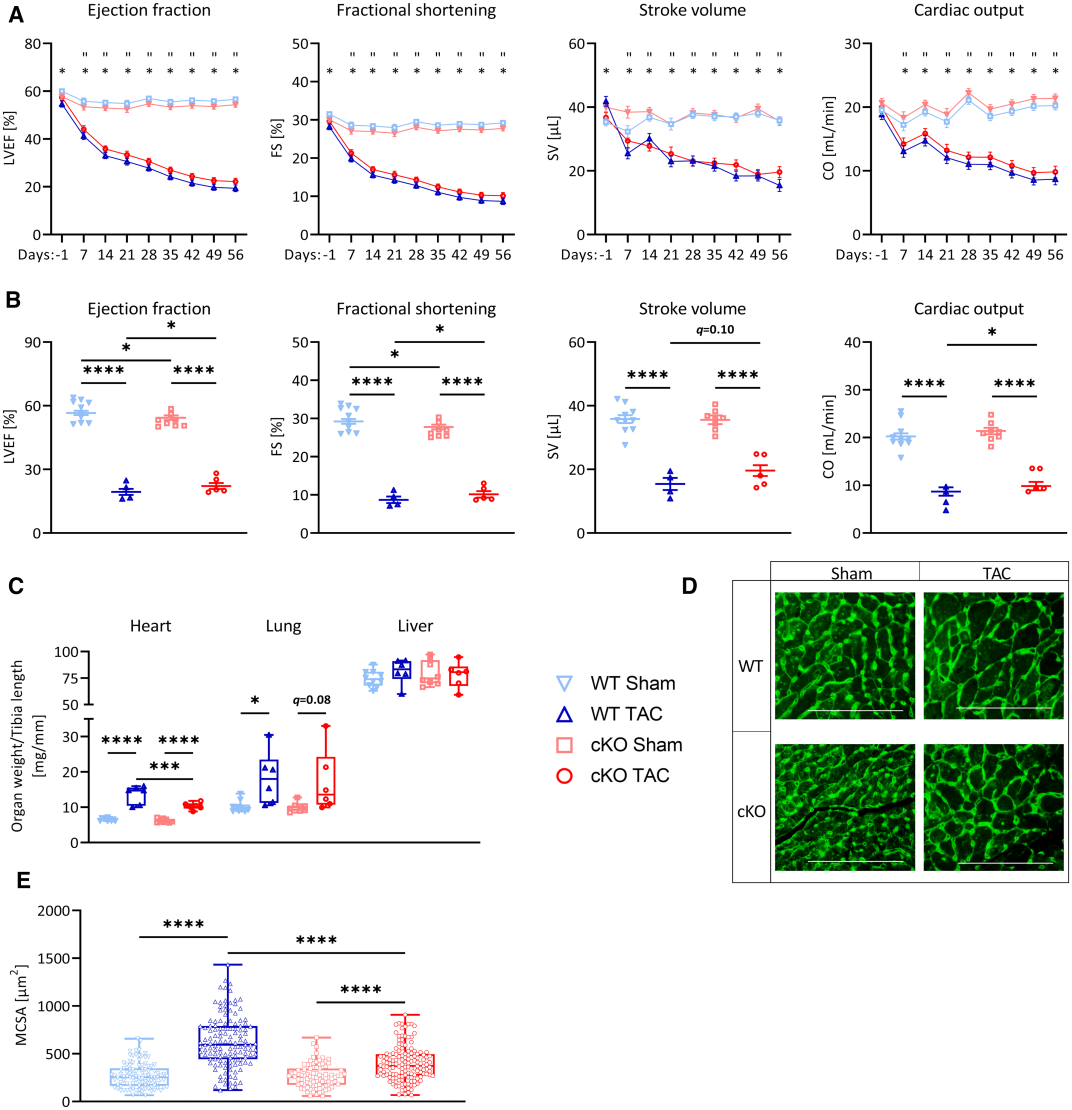
Deletion of TFEB improves cardiac function in pressure overload-induced HFrEF. (**A**) Kinetics of left ventricular ejection fraction (LVEF), fractional shortening (FS), stroke volume (SV), and cardiac output (CO) as determined at indicated time points after Sham and TAC surgery, respectively, of WT and cKO mice (* = WT_TAC/WT_Sham, “ = cKO_TAC/cKO_Sham, & = cKO_TAC/WT_TAC). (**B**) Cardiac function as measured in (**A**) after 56 days of Sham or TAC treated WT and cKO mice. (**C**) Heart, lung, and liver weights of WT and cKO mice after 56 days of Sham or TAC surgery, normalized to tibia length. (**D**) Wheat Germ Agglutinin (WGA) stained histological cross-sections of hearts from WT and cKO mice after 56 days of Sham or TAC surgery. (**E**) Myocyte cross-sectional area (MCSA) measured from on WGA stained sections with Image J. Scale bar, 100 µm. Data are presented as mean ± SD (56 days, WT Sham: *n* = 10, WT TAC: *n* = 6, cKO Sham: *n* = 8, cKO TAC: *n* = 6; ^*,”,&^*q *< 0.05, **q < 0.01, ***q < 0.001, ****q < 0.0001).

**Figure 5:**
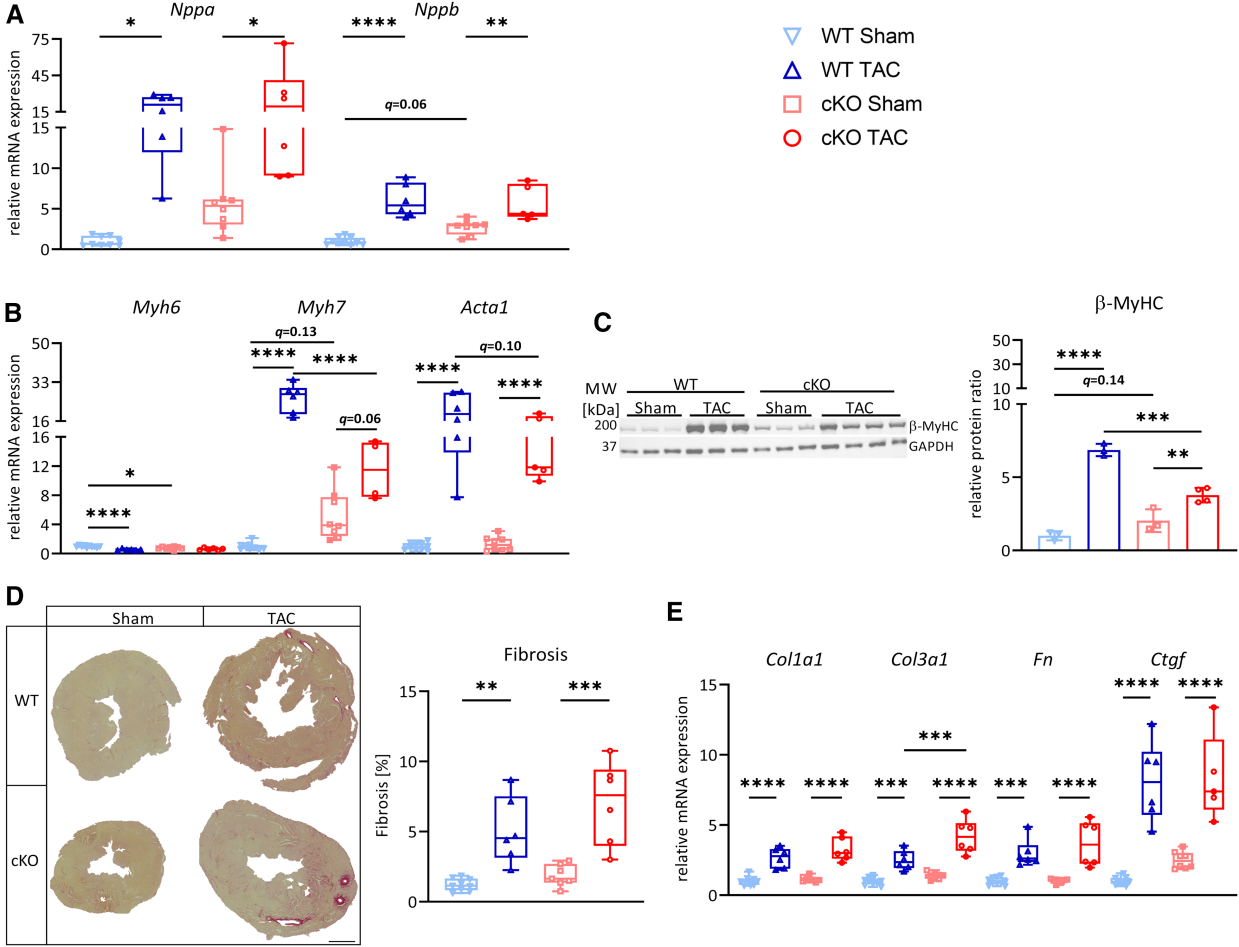
Deletion of TFEB reduces cardiac remodeling in pressure overload-induced HFrEF. Quantitative real-time polymerase chain reaction (qRT-PCR) analysis of *Nppa* and *Nppb* (**A**) and *Myh6*, *Myh7*, and *Acta1* (**B**) expression from WT and cKO mice after 56 days of Sham or TAC surgery as indicated. mRNA expression was normalized to *Gapdh*. (**C**) Western blot analysis with anti-β-MyHC and anti-GAPDH antibodies. GAPDH was used as loading control. Bar graph showing the ratio of the relative densities of β-MyHC and GAPDH protein contents. (**D**) Representative images of Picrosirius Red stained (PSR, left) heart cross-sections of WT and cKO mice after 56 days of Sham or TAC surgery; scale bar, 1 mm. Fibrotic area (right) was measured with Image J. (**E**) qRT-PCR analysis of indicated genes from WT and cKO mice after 56 days of Sham or TAC surgery as indicated. mRNA expression was normalized to *Gapdh*. Data are presented as mean ± SD (56 days, WT Sham: *n* = 10, WT TAC: *n* = 6, cKO Sham: *n* = 8, cKO TAC: *n* = 6; ^*,”,&^*q *< 0.05 **q < 0.01, ***q < 0.001, ****q < 0.0001).

Because cKO mice showed an enhanced cardiac stress response and remodeling after 21 days of TAC without changes in cardiac function, we hypothesized that such effects would occur during PO-induced HFrEF. We subjected 8-weeks old male cKO (Sham: *n* = 8, TAC: *n* = 6) and WT (Sham: *n* = 10, TAC: *n* = 6) mice to 56 days of TAC or Sham surgery. The expression of *Tfeb* increased and *Mitf* decreased in WT TAC compared to WT Sham mice (Supplementary Figure S5A). *Tfe3* expression increased in cKO TAC compared to cKO Sham mice but remained unchanged in WT hearts (Supplementary Figure S5A). Echocardiography revealed a continuous increase in LV mass (Supplementary Figure S5B) and a reduction in LVEF, FS, SV, and CO (Figure 4A; Supplementary Tables S6A,B) in WT TAC and cKO TAC mice compared to their respective Sham group. Cardiac function of cKO TAC mice as assessed by LVEF (2.8%), FS (1.4%), CO (1.14 µl/min) and SV (4.2 µl; trended; *p* = 0.10) was slightly improved in cKO TAC vs. WT TAC mice (Figure 4B; Supplementary Tables S6A,B). cKO TAC mice showed a 24% lower increase in HW/TL ratios (68%) compared to WT TAC (107%) mice (Figure 4C). The Lu/TL ratio was higher in WT TAC compared to WT Sham mice and showed a trend towards an increase in cKO TAC vs. cKO Sham mice (*q *= 0.08, Figure 4C). The Li/TL ratio remained unchanged over all groups (Figure 4C). H&E staining (Supplementary Figure S5C) and MCSA measurements (Figures 4D,E) uncovered cardiomyocyte hypertrophy in WT TAC and cKO TAC mice compared to their respective Sham group. However, this increase was significantly smaller in cKO TAC compared to WT TAC mice (Figure 4E and Supplementary Figures S5D-G). The expression of *Nppa*, *Nppb*, and *Acta1* was increased in WT and cKO TAC mice compared to their Sham groups (Figures 5A,B). The expression of *Myh6* was only decreased in WT but not cKO TAC mice. *Myh7* mRNA expression (WT: *q < *0.0001, cKO: *q = *0.06) and β-MyHC (WT: *q < *0.0001, cKO: *q < *0.01) protein contents were increased in response to TAC in both genotypes. However, this increase was significantly lower in cKO compared to WT TAC mice (Figures 5B,C). PSR staining revealed interstitial fibrosis in WT and cKO TAC mice without differences between the genotypes (Figure 5D), which is in line with the expression of the ECM genes *Col1a1*, *Col3a1*, *Fn,* and *Ctgf* (Figure 5E). In summary, deletion of TFEB in cardiomyocytes is associated with a reduction in cardiac and cardiomyocyte hypertrophy and an improved cardiac function in HFrEF.

### Deletion of TFEB has minor effects on the expression of metabolic remodeling genes in HFrEF

**Figure 6:**
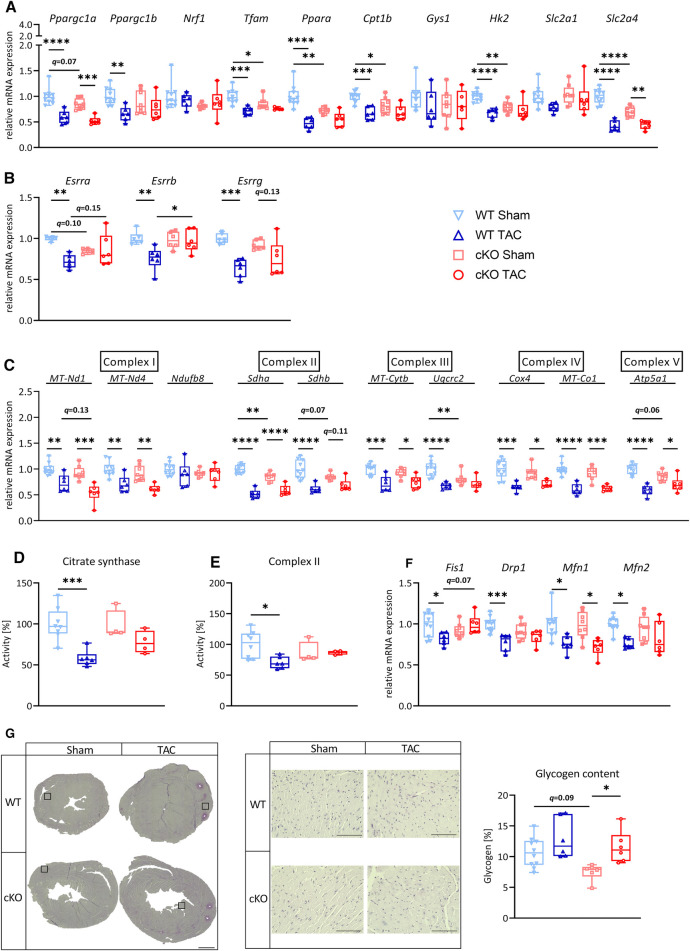
Deletion of TFEB has minor effects on metabolic remodeling and energy homeostasis in HFrEF. Quantitative real-time polymerase chain reaction (qRT-PCR) analysis of indicated metabolic genes (**A–B**) and genes involved in OXPHOS (**C**) and mitochondrial fission and fusion (**F**) from WT and cKO mice after 56days of Sham or TAC surgery as indicated. mRNA expression was normalized to *Gapdh*. (**D**) Citrate synthase and (**E**) complex II activity in protein lysates from hearts of WT and cKO mice after 56 days of Sham or TAC surgery. (**G**) Representative images of PAS stained heart cross-sections of WT and cKO mice after 56 days of Sham or TAC surgery; left: overview, scale bare 1 mm; middle: higher magnification (corresponding to inset from left), scale bar 100 µm, right: quantification of glycogen content with Image J. Data are presented as mean ± SD (56 days, WT Sham: *n* = 10, WT TAC: *n* = 6, cKO Sham: *n* = 8, cKO TAC: *n* = 6; *q < 0.05, **q < 0.01, ***q < 0.001, ****q < 0.0001).

Fifty-six days after surgery, the expression of *Ppargc1a*, *Ppargc1b*, *Ppara*, and *Cpt1b* was reduced in hearts of WT TAC compared to WT Sham mice (Figure 6A), whereas only *Ppargc1a* expression was decreased in hearts of cKO TAC compared to Sham mice. No differences were found between genotypes. *Tfam* was significantly reduced in hearts of WT TAC but not cKO TAC mice compared to their Sham groups. Additionally, *Esrra*, *Esrrb*, and *Esrrg* were significantly lower in hearts of WT TAC but not cKO TAC mice compared to their respective Sham mice (Figure 6B). The expression of most ETC genes, such as *MT-Nd1* (WT: *q *< 0.01, cKO: *q *< 0.001), *MT-Nd4* (WT: *q *< 0.01, cKO: *q *< 0.01), *Sdha* (WT: *q *< 0.0001, cKO: *q *< 0.0001)*, Sdhb* (WT: *q *< 0.0001, cKO: *q *= 0.11), *MT-Cytb* (WT: *q *< 0.001, cKO: *q *< 0.05), *Cox4* (WT: *q *< 0.0001, cKO: *q *< 0.05), *MT-Co1* (WT: *q *< 0.0001, cKO: *q *< 0.001) and *Atp5a1* (WT: *q *< 0.0001, cKO: *q *< 0.05) was reduced in WT TAC and cKO TAC mice compared to their Sham group (Figure 6C). However, only the expression of *Sdha* was significantly lower in cKO TAC compared to WT TAC mice (Figure 6B).

To investigate if the observed changes in mitochondrial gene expression are associated with variations in mitochondrial content, we performed spectrophotometry (40) to measure the activity of citrate synthetase (CS) in protein lysates from hearts of WT and cKO mice. CS activity was lower in WT TAC compared to WT Sham mice, but remained unchanged cKO TAC compared to cKO Sham mice (Figure 6D). We next measured CII activity to evaluate if the reduced *Sdha* expression between WT and cKO mice at baseline and its decrease in response to HFrEF in both genotypes has any effects on CII function. CII activity was comparable between WT Sham and cKO Sham hearts. However, CII activity was reduced in hearts of WT TAC compared to WT Sham mice but remained unchanged in cKO TAC compared to cKO Sham mice (Figure 6E).

The expression of *Fis1*, *Drp1*, *Mfn1*, and *Mfn2* was reduced in WT TAC compared to WT Sham mice. This reduction was not observed in cKO TAC compared to cKO Sham mice except for *Mfn1* (Figure 6F).

*Hk2* and *Slc2a4* were decreased in WT TAC compared to WT Sham and *Gys1* and *Slc2a1* remained unchanged in all groups (Figure 6A). Myocardial glycogen content was higher in cKO TAC, but remained unaffected in WT TAC mice when compared to the respective Sham group (Figure 6G). No differences in glycogen contents were found between cKO TAC and WT TAC mice. In summary, these data indicate that deletion of TFEB in cardiomyocytes has only minor effects on the expression of genes involved in cardiac energy homeostasis in HFrEF.

### The absence of TFEB has only minor effects on the cardiac proteome in response to HFrEF

**Figure 7:**
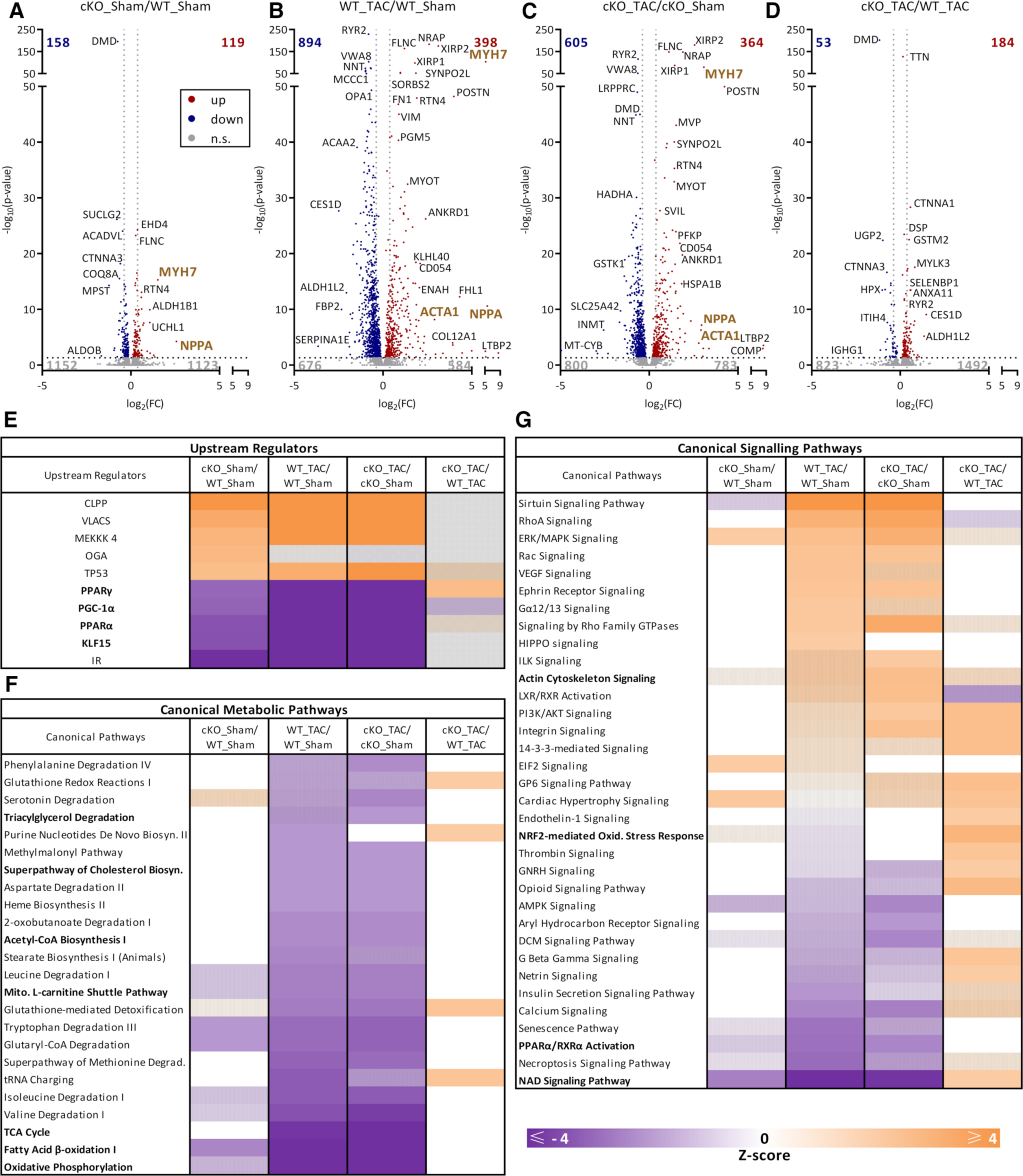
Proteome analysis of hearts from cKO and WT mice in pressure overload-induced HFrEF. (**A–D**) Volcano plots of protein ratios comparing WT Sham, WT TAC, cKO Sham, and cKO TAC as indicated. The number of differentially regulated proteins (DRP) is indicated in the graphs. Proteins that occur at significantly higher (red, *q*-value ≤ 0.05) or lower levels (blue, *q*-value ≤ 0.05) or do not change in abundance [gray, not significant (n.s.)] are indicated. Results of QIAGEN Ingenuity Pathway Analysis (IPA). **(E)** TOP 5 activated and inhibited “upstream regulators”, (**F**) canonical metabolic pathways and (**G**) canonical signaling pathways with significant enrichment of regulated proteins are shown. Data are presented as heat map (*q*-value ≤ 0.05). A positive z-score (≥2) predicts activation (orange), a negative z-score (≤−2) predicts inhibition (purple) of the identified proteins or pathways. Hatched marked areas are not significantly activated or inhibited.

To investigate if the absence of TFEB in cardiomyocytes leads to changes in the cardiac proteome in response to HFrEF we performed mass spectrometric analyses of proteins isolated from cardiac apex of 56-day TAC- and Sham treated WT and cKO mice. Only few proteins were differentially regulated (DRP; 158 down- and 119 up-regulated) between WT Sham and cKO Sham mice (cKO_Sham/WT_Sham; Figure 7A). TAC resulted in pronounced changes in the cardiac proteome of both WT (WT_TAC/WT_Sham: 894 down-, 398 up-regulated; Figure 7B) and cKO (cKO_TAC/cKO_Sham: 605 down-, 364 up-regulated; Figure 7C) mice. However, fewer proteins were differentially regulated and also to a lower degree in cKO TAC when compared to WT TAC mice (cKO_TAC/WT_TAC; Supplementary Figure S6A). For example, ANF (WT TAC: 49.5-fold, cKO TAC: 7.7-fold) and β-MyHC levels (WT TAC: 37.0-fold, cKO TAC: 8.5-fold) were higher in WT TAC compared to cKO TAC mice. Ratios of DRP in WT TAC (WT_TAC/WT_Sham) and cKO TAC (cKO_TAC/cKO_Sham) hearts showed a strong correlation (*r* = 0.928, *p* = 0.0001, Supplementary Figure S6A) supporting the similarity of the TAC effect independent of the genotype (Figure 7D). The scatter plot in Supplementary Figure S6A shows the lower extent of changes in cKO hearts. Correlation analysis of DRP between cKO_TAC/WT_TAC and WT_TAC/WT_Sham indicated that the absence of TFEB partially reversed the TAC effect (*r* = −0.622, *p* = 0.0001, Supplementary Figure S6B), which is in line with our gene expression data. The correlation of DRP between cKO_Sham/WT_Sham and WT_TAC/WT_Sham indicated that the deletion of TFEB on one hand and TAC on the other lead to similar DRP pattern (*r* = 0.7187, *p* = 0.0001, Supplementary Figure S6C) suggesting that the deletion of TFEB *per se* is associated with cardiac stress.

Further analyses of DRP by Ingenuity Pathway Analysis (IPA) uncovered the known TFEB target genes *Ppargc1a*/PGC-1α, *Ppara*/PPARα, and insulin receptor (IR) as well as Krueppel-like factor 15 (KLF15) as potential upstream regulators. The activity of these proteins was predicted as significantly inhibited (z-score < −2) in cKO Sham compared to WT Sham mice (Figure 7E) indicating that TFEB contributes to their baseline expression. Their comparable inhibition in response to TAC in both genotypes (Figure 7E) suggests a minor role for TFEB in HFrEF-induced downregulation of these proteins.

IPA also revealed that the metabolic pathway FAO (z-score < −2.3, Figure 7F) and NAD signaling were inhibited (z-score < −2.5, Figure 7G) and OXPHOS showed a trend towards inhibition (z-score < −1.2) in hearts of cKO Sham compared to WT Sham mice. However, a similar inhibition in FAO (WT: z-score < −4.2, cKO: z-score < −4.2), TCA cycle (WT: z-score < −3.8, cKO: z-score < −4.0), OXPHOS (WT: z-score < −8.1, cKO: z-score < −8.5), mitochondrial L-carnitine shuttle (WT: z-score < −2.4, cKO: z-score < −2.4), Acetyl-CoA Biosynthesis (WT: z-score < −2.2, cKO: z-score < −2.2), and cholesterol biosynthesis (WT: z-score < −2.0, cKO: z-score < −2.0) was observed for WT TAC and cKO TAC hearts compared to their Sham group without differences between genotypes (Figure 7F). IPA analyses of activated and inhibited canonical signaling pathways are shown in Figure 7G. A similar inhibition in PPARα/RXRα activation (WT: z-score < −2.7, cKO: z-score < −2.4) and NAD signaling pathway (WT: z-score < −4.0, cKO: z-score < −3.9) was observed for WT TAC and cKO TAC hearts compared to their Sham group. No difference in PPARα/RXRα activation was observed between genotypes. The NAD signaling pathway displayed a higher activity in cKO TAC (z-score > 2.0) compared to WT TAC mice (Figure 7G). Actin cytoskeleton signaling was only activated in hearts of cKO TAC but not WT TAC mice compared to their respective Sham group. NRF2-mediated oxidative stress response was higher in cKO TAC compared to WT TAC hearts (Figure 7G). The proteins contained within the respective metabolic pathways showed a similar abundance in hearts of WT TAC and cKO TAC mice compared to the respective Sham controls (Supplementary Figures S7A,B). KEGG pathway analyses of proteins contained in FAO (Supplementary Figure S7A) that are reduced in both, WT TAC and cKO TAC, revealed an inhibition of almost all enzymatic steps of this pathway from the fatty acid palmitic acid to acetyl-CoA (Supplementary Figure S7C).

Overall, these proteomics data are in accordance with our gene expression analyses (Supplementary Figures S6D,E) and suggest a metabolic remodeling in response to HFrEF indicative for an inhibition of FAO, OXPHOS, and TCA cycle in the heart. They also show that cardiomyocyte-specific deletion of TFEB inhibits FAO related proteins at baseline but only slightly in HFrEF.

## Discussion

Here we show that the deletion of TFEB in cardiomyocytes is associated with an increased cardiac stress response and remodeling, and affects the expression of genes involved in cardiac energy metabolism, which was associated with a reduced cardiac function of unstressed hearts. In LVH, the loss of TFEB increased cardiac remodeling but did not affect cardiac function. In contrast, deletion of TFEB attenuated cardiac remodeling and improved cardiac function in HFrEF. The deletion of TFEB only slightly affected the expression of metabolic genes in HFrEF hearts. Our data suggest that TFEB has different effects on cardiac remodeling and function in cardiomyocytes under physiological conditions, LVH and HFrEF. They also indicate that TFEB plays only a minor role, if any, in metabolic remodeling that occurs during PO-induced LVH and HFrEF in the heart.

We used TAC-induced PO for 21 days to provoke LVH, which is accompanied by an activation of the fetal gene program, an increased interstitial fibrosis, and a metabolic shift from FAO to GO (6, 25, 56, 57). We also used 56 days of PO to induce HFrEF (31) that is characterized by a decrease in LVEF, LV dilatation, and a reduced LV wall thickness as well as an excessive interstitial and perivascular fibrosis and metabolic remodeling (56, 57). Functional, morphological, and molecular data confirmed that both models were suitable to induce LVH and HFrEF, respectively, with the expected functional, morphological, and molecular changes.

The effects of TFEB on cardiomyocyte stress response were previously investigated by gain- and loss-of-function experiments. Song et al. (2021) showed that a moderate 2-fold over expression of TFEB in the heart of male mice resulted in an improved cardiac function, a reduced fibrosis, and lower expression of cardiac stress markers in response to 56 days of 27G TAC when compared to controls (58). These data indicated that an early activation of TFEB could be useful to prevent pathological cardiac remodeling. Consistent with these data, we found that a deletion of TFEB in cardiomyocytes leads to an increased expression of cardiac stress, remodeling and fibrosis markers and a reduced cardiac function. However, we did not observe a further deterioration in cardiac function in response to PO-induced LVH in cKO mice. In contrast to the results of Song et al. (2021), we recently reported that cardiomyocyte-specific TFEB-overexpression by AAV2.9-mediated gene transfer followed by TAC surgery (27G, 28 days) caused HFrEF whereas control mice developed compensated LVH (22). Specifically, TFEB overexpression was associated with increased heart weights, exaggerated interstitial fibrosis, higher expression of stress markers and remodeling genes in response to TAC (22). In line with our data are findings from Kenny et al. (2021), who showed that TFEB overexpression at higher levels (8-fold increase in TFEB) in cardiomyocytes promoted pathological cardiac hypertrophy, suppressed mitochondrial and activated pro-fibrotic pathways (59). Trivedi et al. (2020) used a loss-of-function approach to investigate the role of TFEB in the heart (60). They found that the absence of TFEB predominantly caused changes in non-canonical TFEB-pathways that are involved in energy metabolism (e.g., lipid metabolism and transport, fatty acid biosynthesis, glycogen metabolism) whereas minor effects were found for canonical TFEB-pathways, such as ALP. In line with our data, they showed that PPARα was decreased in myocytes lacking TFEB, suggesting a decrease in FAO in cKO hearts. They concluded that a loss of TFEB perturbs metabolic pathways in cardiomyocytes and increases the susceptibility of the heart to nutrient overload-induced injury. A further study reported that knockdown of TFEB was accompanied by an increase in ANF and β-MyHC in neonatal rat ventricular cardiomyocytes (NRVCMs) (58). The data of both groups are in line with our observations showing an increase in cardiac stress markers and remodeling genes in unstressed cKO hearts.

To assure a continuous ATP production, the heart can switch between different substrates, such as FFA, glucose, lactate, amino acids, and ketone bodies, and is therefore known as a metabolic omnivore (61). Under physiological conditions the heart predominantly uses FAO to generate ATP (62). Stress-induced changes in substrate utilization from FAO to GO is known as metabolic shift (63). This metabolic shift is mediated by multiple factors (7), but transcriptional regulators like PPARα, PGC-1α, and ERRα are primarily involved (14, 52, 64). PPARα increases the expression of genes related to FA uptake and FAO, and is highly expressed in the myocardium. PGC-1α and PGC-1β are essential regulators of mitochondrial biogenesis but require co-activating PPARs or ERR to function properly (52, 53, 65). In HFrEF, FAO decreases (13, 66) and glucose uptake and glycolytic rates increase, which is not accompanied by a concomitant increase in GO (66–68). The shift from FAO to GO is accompanied by a down-regulation of FAO enzymes but the FA uptake remains unchanged. The disturbed FAO together with a continuous FA uptake causes lipid accumulation in cardiomyocytes that have lipotoxic properties (69, 70) and cause mitochondrial dysfunction and apoptosis contributing to progression of HFrEF (71, 72). In the later stages of HFrEF myocardial insulin sensitivity is reduced (73, 74), which impairs cardiac glucose uptake and subsequent ATP production (75). Therefore, the decline in FAO cannot be compensated by GO in HFrEF. We here show an increase in myocardial glycogen content in response to PO, suggestive for a perturbed glucose utilization. We also observed changes in gene expression indicative for a metabolic shift and a severe impairment of energy homeostasis in our experimental models, revealing an inhibition of FAO, TCA cycle, and OXPHOS. In hearts of HFrEF mice, this was accompanied by a suppression of the PPARα/RXRα pathway. As a nutrient-sensitive transcription factor, TFEB increases the expression of PPARα, PGC-1α, and ERRα and can thus regulate FAO and OXPHOS (55, 76). In turn, an increase in PGC-1α activates *Nrf1* and its downstream target *Tfam* to stimulate mitochondrial biogenesis and mitochondrial energy metabolism (51). Accordingly, the deletion of TFEB in cardiomyocytes was shown to reduce PPARα expression and to impair FA utilization (60). These data are in line with our observation that the expression of genes involved in mitochondrial biogenesis (i.e., *Ppargc1a*, *Nrf1*, *Tfam*), FAO (i.e., *Ppara*, *Cpt1b*) and OXPHOS (i.e., *Sdha, Sdhb*, *Uqcrc2*, *Atp5a1*) was reduced in hearts of cKO mice. Our proteomics analyses showed an inhibition of FAO related proteins in hearts of cKO mice and thus also support these observations. These data and previously published work (22, 58) suggest an involvement of TFEB in stress-induced metabolic remodeling during LVH and HFrEF. Although, PGC-1α has been shown to be involved in regulation of mitochondrial quality control we did not observe any differences in the expression of *Fis1*, *Drp1*, *Mfn1* and *Mfn2* that are associated with mitochondrial fission and fusion (47) in hearts of cKO mice. However, further studies are needed to investigate possible changes in this pathway in response to TFEB deletion and myocardial stress. The reduction in *Ppara* and *Cpt1b* expression, as well as ETC subunits in hearts of WT LVH and WT HFrEF mice pointed towards a metabolic shift in response to stress. However, only few (LVH) or no (HFrEF) differences for genes and proteins related to mitochondrial biogenesis, energy homeostasis and OXPHOS were found between WT and cKO mice. These data indicate that the absence of TFEB in cardiomyocytes has only minor effects on proteins involved in metabolic remodeling, mitochondrial biogenesis, energy homeostasis, and OXPHOS in LVH and HFrEF.

Although a switch from FAO to GO occurs in response to cardiac stress it does not mean that this shift is pathologic. In fact, a switch from FAO to GO can also be beneficial under certain circumstances, such as ischemia/reperfusion (77). Under physiological conditions the heart uses FFA over glucose as fuel. FFA through FAO provide the majority of ATP for cardiomyocytes. Although FAO generates more ATP molecules the GO-dependent ATP production rate is faster. This could be important during stress situations when a higher and continuous ATP supply is needed. Also, FAO needs more oxygen for ATP production than GO, which becomes meaningful for the heart during PO where left ventricular wall tension that reduces myocardial perfusion is increased or myocardial ischemia where perfusion in general is limited. In fact, it has been shown that a switch to GO can be beneficial in reperfusion injury (77). Under stress FAO is also associated with an increased production of toxic lipid intermediates and reactive oxygen species (ROS), which may cause lipotoxicity and mitochondrial damage (10), respectively. In contrast, GO does not cause accumulation of toxic lipid intermediates and is associated with lower ROS production, which could be beneficial for mitochondrial as well as cardiomyocyte function. Therefore, a switch from FAO to GO may also have favorable effects for the heart.

Under physiological conditions, the deletion of TFEB was accompanied by an increased expression of stress markers, cardiac remodeling, a decreased cardiac function, and downregulation of genes involved in energy metabolism and ETC. We therefore hypothesized that deletion of TFEB would increase the susceptibility of the heart to LVH and HFrEF, which however could not be confirmed. The MiT/TFE family of transcription factors recognizes unique E-box motifs within the proximal promoters of its target genes and regulates cellular catabolism and nutrient-dependent lysosomal activity (24, 48). TFEB and TFE3 have partially redundant functions, and activate the ALP in non-myocytes (78–80). Pastore et al. (2017) showed that both overexpression of TFEB in *Tfe3*-KO mice and overexpression of TFE3 in liver-specific *Tfeb* cKO mice reversed high-fat diet-induced obesity. This experiment illustrated partially redundant functions of TFEB and TFE3 (50). Because TFE3 has many overlapping functions with TFEB (50, 81), we assume that TFE3 can compensate for the loss of TFEB (82) in cardiomyocytes and that the deletion of both TFEB and TFE3 is necessary to obtain a noticeable phenotype in LVH and HFrEF. This is supported by our observation that both TFEB and TFE3 increase the expression of *PPARGC1A*/PGC-1α and that the activity of both transcription factors is controlled by class IIa HDACs and the protein kinase D family (23, 24). Although the expression of *Tfe3* was reduced or unchanged under physiological conditions and LVH, it was increased in HFrEF cKO but not in HFrEF WT mice. Interestingly, a 24% lower heart weight, a reduced LV mass and a smaller myocyte size were observed in HFrEF cKO mice when compared to WT mice. This was paralleled by a slightly improved cardiac function (LVEF, FS, and CO) and a weaker increase in β-MyHC mRNA and protein level in HFrEF cKO mice. Whether this phenotype is related to the compensatory increase in TFE3 expression is not clear. In summary, our observations suggest that TFE3 may compensate for the loss of TFEB in response to PO-induced HFrEF in *Tfeb* cKO mice. However, this hypothesis warrants further investigation.

## Limitations

Pressure-overload is frequently used to induce pathological LVH and interstitial remodeling in rodents (3, 83). The degree of these responses depends on the severity of aortic constriction and its duration (3). TAC is also used to persistently increased afterload and model the transition from LVH to HFrEF (31). We have recently shown that 28 days of a 27G TAC leads to LVH (32) and that 56 days of 27G TAC causes LVH that transitions into HFrEF (31). In an treatment approach, we used these models to confirm that the soluble guanylyl cyclase activator Riociguat is effective to treat PO-induced HFrEF and the accompanying myocardial remodeling (32). Due to the different timepoints (21 days of TAC (LVH) vs. 56 days of TAC (HFrEF)) it is difficult to compare molecular changes between these two cardiac stress models. However, because our primary aim was to assess the effects of TFEB deficiency in cardiomyocytes in response to PO-induced LVH and HFrEF, respectively, we think that the comparisons of changes in gene expression patterns within each model are also informative. Although a higher afterload could have been chosen to induce HFrEF after 28 days (i.e., 28G TAC) we used a longer time frame to provoke HFrEF as this model shows a phase of LVH; reflecting the transition of LVH to HFrEF, which may be comparable to patients with long term arterial hypertension or aortic valve stenosis.

We here investigated changes in gene expression and contents of proteins that are important for myocardial energy supply and that play a role in GO, FAO and OXPHOS. However, analyses of enzyme activities, metabolite levels and metabolic flux assays are needed to functionally characterize the metabolic shift in LVH and HFrEF.

## Data Availability

The datasets presented in this study can be found in online repositories. The names of the repository/repositories and accession number(s) can be found in the article/**Supplementary Material**.

## References

[1] Savarese G Becher PM Lund LH Seferovic P Rosano GMC Coats AJS. Global burden of heart failure: a comprehensive and updated review of epidemiology. Cardiovasc Res. (2023) 118(17):3272–87. 10.1093/cvr/cvac013 35150240

[2] Nakamura M Sadoshima J. Mechanisms of physiological and pathological cardiac hypertrophy. Nat Rev Cardiol. (2018) 15(7):387–407. 10.1038/s41569-018-0007-y 29674714

[3] Melleby AO Romaine A Aronsen JM Veras I Zhang L Sjaastad I A novel method for high precision aortic constriction that allows for generation of specific cardiac phenotypes in mice. Cardiovasc Res. (2018) 114(12):1680–90. 10.1093/cvr/cvy141 29878127

[4] Kattih B Boeckling F Shumliakivska M Tombor L Rasper T Schmitz K Single-nuclear transcriptome profiling identifies persistent fibroblast activation in hypertrophic and failing human hearts of patients with longstanding disease. Cardiovasc Res. (2023) 119(15):2550–62. 10.1093/cvr/cvad140 37648651

[5] Halliday BP Prasad SK. The interstitium in the hypertrophied heart. JACC Cardiovasc Imaging. (2019) 12(11 Pt 2):2357–68. 10.1016/j.jcmg.2019.05.033 31542527

[6] Fielitz J Hein S Mitrovic V Pregla R Zurbrügg HR Warnecke C Activation of the cardiac renin-angiotensin system and increased myocardial collagen expression in human aortic valve disease. J Am Coll Cardiol. (2001) 37(5):1443–9. 10.1016/S0735-1097(01)01170-6 11300459

[7] Bertero E Maack C. Metabolic remodelling in heart failure. Nat Rev Cardiol. (2018) 15(8):457–70. 10.1038/s41569-018-0044-6 29915254

[8] Lopaschuk GD Ussher JR Folmes CD Jaswal JS Stanley WC. Myocardial fatty acid metabolism in health and disease. Physiol Rev. (2010) 90(1):207–58. 10.1152/physrev.00015.2009 20086077

[9] Lopaschuk GD Karwi QG Tian R Wende AR Abel ED. Cardiac energy metabolism in heart failure. Circ Res. (2021) 128(10):1487–513. 10.1161/CIRCRESAHA.121.318241 33983836 PMC8136750

[10] Abdurrachim D Luiken JJ Nicolay K Glatz JF Prompers JJ Nabben M. Good and bad consequences of altered fatty acid metabolism in heart failure: evidence from mouse models. Cardiovasc Res. (2015) 106(2):194–205. 10.1093/cvr/cvv105 25765936

[11] Oka SI Sabry AD Cawley KM Warren JS. Multiple levels of PGC-1alpha dysregulation in heart failure. Front Cardiovasc Med. (2020) 7:2. 10.3389/fcvm.2020.00002 32083094 PMC7002390

[12] Karwi QG Uddin GM Ho KL Lopaschuk GD. Loss of metabolic flexibility in the failing heart. Front Cardiovasc Med. (2018) 5:68. 10.3389/fcvm.2018.00068 29928647 PMC5997788

[13] Doenst T Nguyen TD Abel ED. Cardiac metabolism in heart failure: implications beyond ATP production. Circ Res. (2013) 113(6):709–24. 10.1161/CIRCRESAHA.113.300376 23989714 PMC3896379

[14] Lefebvre P Chinetti G Fruchart JC Staels B. Sorting out the roles of PPAR alpha in energy metabolism and vascular homeostasis. J Clin Invest. (2006) 116(3):571–80. 10.1172/JCI27989 16511589 PMC1386122

[15] Huss JM Kelly DP. Mitochondrial energy metabolism in heart failure: a question of balance. J Clin Invest. (2005) 115(3):547–55. 10.1172/JCI24405 15765136 PMC1052011

[16] Lehman JJ Kelly DP. Transcriptional activation of energy metabolic switches in the developing and hypertrophied heart. Clin Exp Pharmacol Physiol. (2002) 29(4):339–45. 10.1046/j.1440-1681.2002.03655.x 11985547

[17] Panagia M Gibbons GF Radda GK Clarke K. PPAR-alpha activation required for decreased glucose uptake and increased susceptibility to injury during ischemia. Am J Physiol Heart Circ Physiol. (2005) 288(6):H2677–83. 10.1152/ajpheart.00200.2004 15665064

[18] Campbell FM Kozak R Wagner A Altarejos JY Dyck JR Belke DD A role for peroxisome proliferator-activated receptor alpha (PPARalpha) in the control of cardiac malonyl-CoA levels: reduced fatty acid oxidation rates and increased glucose oxidation rates in the hearts of mice lacking PPARalpha are associated with higher concentrations of malonyl-CoA and reduced expression of malonyl-CoA decarboxylase. J Biol Chem. (2002) 277(6):4098–103. 10.1074/jbc.M106054200 11734553

[19] Settembre C De Cegli R Mansueto G Saha PK Vetrini F Visvikis O TFEB controls cellular lipid metabolism through a starvation-induced autoregulatory loop. Nat Cell Biol. (2013) 15(6):647–58. 10.1038/ncb2718 23604321 PMC3699877

[20] Mansueto G Armani A Viscomi C D'Orsi L De Cegli R Polishchuk EV Transcription factor EB controls metabolic flexibility during exercise. Cell Metab. (2017) 25(1):182–96. 10.1016/j.cmet.2016.11.003 28011087 PMC5241227

[21] Wang S Chen Y Li X Zhang W Liu Z Wu M Emerging role of transcription factor EB in mitochondrial quality control. Biomed Pharmacother. (2020) 128:110272. 10.1016/j.biopha.2020.110272 32447212

[22] Wundersitz S Pablo Tortola C Schmidt S Oliveira Vidal R Kny M Hahn A The transcription factor EB (TFEB) sensitizes the heart to chronic pressure overload. Int J Mol Sci. (2022) 23(11):5943. 10.3390/ijms23115943 35682624 PMC9180101

[23] Tortola C P Fielitz B Li Y Rudebusch J Luft FC Fielitz J. Activation of tripartite motif containing 63 expression by transcription factor EB and transcription factor binding to immunoglobulin heavy chain enhancer 3 is regulated by protein kinase D and class IIa histone deacetylases. Front Physiol. (2020) 11:550506. 10.3389/fphys.2020.550506 33519497 PMC7838639

[24] Du Bois P Pablo Tortola C Lodka D Kny M Schmidt F Song K Angiotensin II induces skeletal muscle atrophy by activating TFEB-mediated MuRF1 expression. Circ Res. (2015) 117(5):424–36. 10.1161/CIRCRESAHA.114.305393 26137861 PMC4537692

[25] Fielitz J Kim MS Shelton JM Qi X Hill JA Richardson JA Requirement of protein kinase D1 for pathological cardiac remodeling. Proc Natl Acad Sci U S A. (2008) 105(8):3059–63. 10.1073/pnas.0712265105 18287012 PMC2268584

[26] Weeks KL Avkiran M. Roles and post-translational regulation of cardiac class IIa histone deacetylase isoforms. J Physiol. (2015) 593(8):1785–97. 10.1113/jphysiol.2014.282442 25362149 PMC4405742

[27] Hohl M Wagner M Reil JC Muller SA Tauchnitz M Zimmer AM HDAC4 controls histone methylation in response to elevated cardiac load. J Clin Invest. (2013) 123(3):1359–70. 10.1172/JCI61084 23434587 PMC3582114

[28] Zhang CL McKinsey TA Chang S Antos CL Hill JA Olson EN. Class II histone deacetylases act as signal-responsive repressors of cardiac hypertrophy. Cell. (2002) 110(4):479–88. 10.1016/S0092-8674(02)00861-9 12202037 PMC4459650

[29] Chang S McKinsey TA Zhang CL Richardson JA Hill JA Olson EN. Histone deacetylases 5 and 9 govern responsiveness of the heart to a subset of stress signals and play redundant roles in heart development. Mol Cell Biol. (2004) 24(19):8467–76. 10.1128/MCB.24.19.8467-8476.2004 15367668 PMC516756

[30] Agah R Frenkel PA French BA Michael LH Overbeek PA Schneider MD. Gene recombination in postmitotic cells. Targeted expression of cre recombinase provokes cardiac-restricted, site-specific rearrangement in adult ventricular muscle in vivo. J Clin Invest. (1997) 100(1):169–79. 10.1172/JCI119509 9202069 PMC508177

[31] Rudebusch J Benkner A Poesch A Dorr M Volker U Grube K Dynamic adaptation of myocardial proteome during heart failure development. PLoS One. (2017) 12(10):e0185915. 10.1371/journal.pone.0185915 28973020 PMC5626523

[32] Rudebusch J Benkner A Nath N Fleuch L Kaderali L Grube K Stimulation of soluble guanylyl cyclase (sGC) by riociguat attenuates heart failure and pathological cardiac remodelling. Br J Pharmacol. (2022). 179(11):2430–42. 10.1111/bph.15333 33247945

[33] Lodka D Pahuja A Geers-Knorr C Scheibe RJ Nowak M Hamati J Muscle RING-finger 2 and 3 maintain striated-muscle structure and function. J Cachexia Sarcopenia Muscle. (2016) 7(2):165–80. 10.1002/jcsm.12057 27493870 PMC4863828

[34] Fielitz J van Rooij E Spencer JA Shelton JM Latif S van der Nagel R Loss of muscle-specific RING-finger 3 predisposes the heart to cardiac rupture after myocardial infarction. Proc Natl Acad Sci U S A. (2007) 104(11):4377–82. 10.1073/pnas.0611726104 17360532 PMC1838610

[35] Fielitz J Kim MS Shelton JM Latif S Spencer JA Glass DJ Myosin accumulation and striated muscle myopathy result from the loss of muscle RING finger 1 and 3. J Clin Invest. (2007) 117(9):2486–95. 10.1172/JCI32827 17786241 PMC1957544

[36] Hahn A Kny M Pablo-Tortola C Todiras M Willenbrock M Schmidt S Serum amyloid A1 mediates myotube atrophy via toll-like receptors. J Cachexia Sarcopenia Muscle. (2020) 11(1):103–19. 10.1002/jcsm.12491 31441598 PMC7015249

[37] Zanders L Kny M Hahn A Schmidt S Wundersitz S Todiras M Sepsis induces interleukin 6, gp130/JAK2/STAT3, and muscle wasting. J Cachexia Sarcopenia Muscle. (2022) 13(1):713–27. 10.1002/jcsm.12867 34821076 PMC8818599

[38] Kny M Csalyi KD Klaeske K Busch K Meyer AM Merks AM Ninjurin1 regulates striated muscle growth and differentiation. PLoS One. (2019) 14(5):e0216987. 10.1371/journal.pone.0216987 31091274 PMC6519837

[39] Busch K Kny M Huang N Klassert TE Stock M Hahn A Inhibition of the NLRP3/IL-1beta axis protects against sepsis-induced cardiomyopathy. J Cachexia Sarcopenia Muscle. (2021) 12(6):1653–68. 10.1002/jcsm.12763 34472725 PMC8718055

[40] Spinazzi M Casarin A Pertegato V Salviati L Angelini C. Assessment of mitochondrial respiratory chain enzymatic activities on tissues and cultured cells. Nat Protoc. (2012) 7(6):1235–46. 10.1038/nprot.2012.058 22653162

[41] Vega RB Harrison BC Meadows E Roberts CR Papst PJ Olson EN Protein kinases C and D mediate agonist-dependent cardiac hypertrophy through nuclear export of histone deacetylase 5. Mol Cell Biol. (2004) 24(19):8374–85. 10.1128/MCB.24.19.8374-8385.2004 15367659 PMC516754

[42] Kim YS Lee HM Kim JK Yang CS Kim TS Jung M PPAR-alpha activation mediates innate host defense through induction of TFEB and lipid catabolism. J Immunol. (2017) 198(8):3283–95. 10.4049/jimmunol.1601920 28275133

[43] Blankenburg S Hentschker C Nagel A Hildebrandt P Michalik S Dittmar D Improving proteome coverage for small sample amounts: an advanced method for proteomics approaches with low bacterial cell numbers. Proteomics. (2019) 19(23):e1900192. 10.1002/pmic.201900192 31532911

[44] Suomi T Elo LL. Enhanced differential expression statistics for data-independent acquisition proteomics. Sci Rep. (2017) 7(1):5869. 10.1038/s41598-017-05949-y 28724900 PMC5517573

[45] Benjamini Y Krieger AM Yekutieli D. Adaptive linear step-up procedures that control the false discovery rate. Biometrika. (2006) 93(3):491–507. 10.1093/biomet/93.3.491

[46] Dabrowska A Venero JL Iwasawa R Hankir MK Rahman S Boobis A PGC-1alpha controls mitochondrial biogenesis and dynamics in lead-induced neurotoxicity. Aging (Albany NY). (2015) 7(9):629–47. 10.18632/aging.100790 26363853 PMC4600622

[47] Peng K Yang L Wang J Ye F Dan G Zhao Y The interaction of mitochondrial biogenesis and fission/fusion mediated by PGC-1alpha regulates rotenone-induced dopaminergic neurotoxicity. Mol Neurobiol. (2017) 54(5):3783–97. 10.1007/s12035-016-9944-9 27271125

[48] Lu H Sun J Hamblin MH Chen YE Fan Y. Transcription factor EB regulates cardiovascular homeostasis. EBioMedicine. (2021) 63:103207. 10.1016/j.ebiom.2020.103207 33418500 PMC7804971

[49] Pastore N Brady OA Diab HI Martina JA Sun L Huynh T TFEB and TFE3 cooperate in the regulation of the innate immune response in activated macrophages. Autophagy. (2016) 12(8):1240–58. 10.1080/15548627.2016.1179405 27171064 PMC4968228

[50] Pastore N Vainshtein A Klisch TJ Armani A Huynh T Herz NJ TFE3 regulates whole-body energy metabolism in cooperation with TFEB. EMBO Mol Med. (2017) 9(5):605–21. 10.15252/emmm.201607204 28283651 PMC5412821

[51] Gonçalves VF. Chapter 3—DNA transcription and translation in mitochondria. In: de Oliveira MR, editor. *Mitochondrial physiology and vegetal molecules*. Academic Press (2021). p. 91–104. 10.1016/B978-0-12-821562-3.00026-5

[52] Sakamoto T Matsuura TR Wan S Ryba DM Kim JU Won KJ A critical role for estrogen-related receptor signaling in cardiac maturation. Circ Res. (2020) 126(12):1685–702. 10.1161/CIRCRESAHA.119.316100 32212902 PMC7274895

[53] Lasheras J Pardo R Velilla M Poncelas M Salvatella N Simo R Cardiac-specific overexpression of ERRgamma in mice induces severe heart dysfunction and early lethality. Int J Mol Sci. (2021) 22(15):8047. 10.3390/ijms22158047 34360813 PMC8348522

[54] Schreiber SN Knutti D Brogli K Uhlmann T Kralli A. The transcriptional coactivator PGC-1 regulates the expression and activity of the orphan nuclear receptor estrogen-related receptor alpha (ERRalpha). J Biol Chem. (2003) 278(11):9013–8. 10.1074/jbc.M212923200 12522104

[55] Malik N Ferreira BI Hollstein PE Curtis SD Trefts E Weiser Novak S Induction of lysosomal and mitochondrial biogenesis by AMPK phosphorylation of FNIP1. Science. (2023) 380(6642):eabj5559. 10.1126/science.abj5559 37079666 PMC10794112

[56] Tham YK Bernardo BC Ooi JY Weeks KL McMullen JR. Pathophysiology of cardiac hypertrophy and heart failure: signaling pathways and novel therapeutic targets. Arch Toxicol. (2015) 89(9):1401–38. 10.1007/s00204-015-1477-x 25708889

[57] Lazzeroni D Rimoldi O Camici PG. From left ventricular hypertrophy to dysfunction and failure. Circ J. (2016) 80(3):555–64. 10.1253/circj.CJ-16-0062 26853555

[58] Song Lei H Feng L Cheng W Li Y Yao LL TFEB insufficiency promotes cardiac hypertrophy by blocking autophagic degradation of GATA4. J Biol Chem. (2021) 297(4):101189. 10.1016/j.jbc.2021.101189 34517007 PMC8498468

[59] Kenny HC Weatherford ET Collins GV Allamargot C Gesalla T Zimmerman K Cardiac specific overexpression of transcription factor EB (TFEB) in normal hearts induces pathologic cardiac hypertrophy and lethal cardiomyopathy. bioRxiv. (2021):2021.02.16.431474. 10.1101/2021.02.16.431474

[60] Trivedi PC Bartlett JJ Mercer A Slade L Surette M Ballabio A Loss of function of transcription factor EB remodels lipid metabolism and cell death pathways in the cardiomyocyte. Biochim Biophys Acta Mol Basis Dis. (2020) 1866(10):165832. 10.1016/j.bbadis.2020.165832 32437957

[61] Taegtmeyer H Golfman L Sharma S Razeghi P van Arsdall M. Linking gene expression to function: metabolic flexibility in the normal and diseased heart. Ann N Y Acad Sci. (2004) 1015:202–13. 10.1196/annals.1302.017 15201161

[62] Stanley WC Recchia FA Lopaschuk GD. Myocardial substrate metabolism in the normal and failing heart. Physiol Rev. (2005) 85(3):1093–129. 10.1152/physrev.00006.2004 15987803

[63] Goodwin GW Taylor CS Taegtmeyer H. Regulation of energy metabolism of the heart during acute increase in heart work. J Biol Chem. (1998) 273(45):29530–9. 10.1074/jbc.273.45.29530 9792661

[64] Cheng CF Ku HC Lin H. PGC-1alpha as a pivotal factor in lipid and metabolic regulation. Int J Mol Sci. (2018) 19(11):3447. 10.3390/ijms19113447 30400212 PMC6274980

[65] Lehman JJ Barger PM Kovacs A Saffitz JE Medeiros DM Kelly DP. Peroxisome proliferator-activated receptor gamma coactivator-1 promotes cardiac mitochondrial biogenesis. J Clin Invest. (2000) 106(7):847–56. 10.1172/JCI10268 11018072 PMC517815

[66] Kolwicz SC Jr Purohit S Tian R. Cardiac metabolism and its interactions with contraction, growth, and survival of cardiomyocytes. Circ Res. (2013) 113(5):603–16. 10.1161/CIRCRESAHA.113.302095 23948585 PMC3845521

[67] Pound KM Sorokina N Ballal K Berkich DA Fasano M Lanoue KF Substrate-enzyme competition attenuates upregulated anaplerotic flux through malic enzyme in hypertrophied rat heart and restores triacylglyceride content: attenuating upregulated anaplerosis in hypertrophy. Circ Res. (2009) 104(6):805–12. 10.1161/CIRCRESAHA.108.189951 19213957 PMC2908318

[68] Sorokina N O'Donnell JM McKinney RD Pound KM Woldegiorgis G LaNoue KF Recruitment of compensatory pathways to sustain oxidative flux with reduced carnitine palmitoyltransferase I activity characterizes inefficiency in energy metabolism in hypertrophied hearts. Circulation. (2007) 115(15):2033–41. 10.1161/CIRCULATIONAHA.106.668665 17404155

[69] Sharma S Adrogue JV Golfman L Uray I Lemm J Youker K Intramyocardial lipid accumulation in the failing human heart resembles the lipotoxic rat heart. FASEB J. (2004) 18(14):1692–700. 10.1096/fj.04-2263com 15522914

[70] Krishnan J Suter M Windak R Krebs T Felley A Montessuit C Activation of a HIF1alpha-PPARgamma axis underlies the integration of glycolytic and lipid anabolic pathways in pathologic cardiac hypertrophy. Cell Metab. (2009) 9(6):512–24. 10.1016/j.cmet.2009.05.005 19490906

[71] Goldberg IJ Trent CM Schulze PC. Lipid metabolism and toxicity in the heart. Cell Metab. (2012) 15(6):805–12. 10.1016/j.cmet.2012.04.006 22682221 PMC3387529

[72] Wende AR Abel ED. Lipotoxicity in the heart. Biochim Biophys Acta. (2010) 1801(3):311–9. 10.1016/j.bbalip.2009.09.023 19818871 PMC2823976

[73] Zhang L Jaswal JS Ussher JR Sankaralingam S Wagg C Zaugg M Cardiac insulin-resistance and decreased mitochondrial energy production precede the development of systolic heart failure after pressure-overload hypertrophy. Circ Heart Fail. (2013) 6(5):1039–48. 10.1161/CIRCHEARTFAILURE.112.000228 23861485

[74] Mori J Alrob OA Wagg CS Harris RA Lopaschuk GD Oudit GY. ANG II causes insulin resistance and induces cardiac metabolic switch and inefficiency: a critical role of PDK4. Am J Physiol Heart Circ Physiol. (2013) 304(8):H1103–13. 10.1152/ajpheart.00636.2012 23396452

[75] Swan JW Anker SD Walton C Godsland IF Clark AL Leyva F Insulin resistance in chronic heart failure: relation to severity and etiology of heart failure. J Am Coll Cardiol. (1997) 30(2):527–32. 10.1016/S0735-1097(97)00185-X 9247528

[76] Pastore N Vainshtein A Herz NJ Huynh T Brunetti L Klisch TJ Nutrient-sensitive transcription factors TFEB and TFE3 couple autophagy and metabolism to the peripheral clock. EMBO J. (2019) 38(12):e101347. 10.15252/embj.2018101347 31126958 PMC6576167

[77] Oeing CU Jun S Mishra S Dunkerly-Eyring BL Chen A Grajeda MI MTORC1-regulated metabolism controlled by TSC2 limits cardiac reperfusion injury. Circ Res. (2021) 128(5):639–51. 10.1161/CIRCRESAHA.120.317710 33401933 PMC8257748

[78] Sardiello M Palmieri M di Ronza A Medina DL Valenza M Gennarino VA A gene network regulating lysosomal biogenesis and function. Science. (2009) 325(5939):473–7. 10.1126/science.1174447 19556463

[79] Settembre C Di Malta C Polito VA Garcia Arencibia M Vetrini F Erdin S TFEB links autophagy to lysosomal biogenesis. Science. (2011) 332(6036):1429–33. 10.1126/science.1204592 21617040 PMC3638014

[80] Settembre C Fraldi A Medina DL Ballabio A. Signals from the lysosome: a control centre for cellular clearance and energy metabolism. Nat Rev Mol Cell Biol. (2013) 14(5):283–96. 10.1038/nrm3565 23609508 PMC4387238

[81] Martina JA Diab HI Lishu L Jeong AL Patange S Raben N The nutrient-responsive transcription factor TFE3 promotes autophagy, lysosomal biogenesis, and clearance of cellular debris. Sci Signal. (2014) 7(309):ra9. 10.1126/scisignal.2004754 24448649 PMC4696865

[82] La Spina M Contreras PS Rissone A Meena NK Jeong E Martina JA. MiT/TFE family of transcription factors: an evolutionary perspective. Front Cell Dev Biol. (2020) 8:609683. 10.3389/fcell.2020.609683 33490073 PMC7815692

[83] Mohammed SF Storlie JR Oehler EA Bowen LA Korinek J Lam CS Variable phenotype in murine transverse aortic constriction. Cardiovasc Pathol. (2012) 21(3):188–98. 10.1016/j.carpath.2011.05.002 21764606 PMC3412352

