# Supplementary material for: Metabolic remodeling in cardiac hypertrophy and heart failure with reduced ejection fraction occurs independent of transcription factor EB in mice
Source: Front Cardiovasc Med. 2024 Jan 8;10:1323760. doi: 10.3389/fcvm.2023.1323760 (PMC10800928; doi:10.3389/fcvm.2023.1323760)
Supplement: Supplementary file 3 [file Datasheet1.pdf]

Supplementary Material

Metabolic remodeling in cardiac hypertrophy and heart failure with reduced ejection fraction occurs independent of transcription factor EB in mice

Niklas Dörmann, Elke Hammer, Karlotta Struckmann, Julia Rüdebusch, Kirsten Bartels, Kristin Wenzel, Julia Schulz, Stefan Gross, Stefan Schwanz, Elisa Martin, Britta Fielitz, Cristina Pablo Tortola, Alexander Hahn, Alexander Benkner, Uwe Völker, Stephan B. Felix, Jens Fielitz

1 Supplementary Figures and Tables

1.1 Supplementary Figures

Supplementary Figure 1. TFE3-induced *Ppargc1a* expression is regulated by class IIa HDACs and PKD. (**A**) Luciferase assays performed with cell extracts of COS-7 cells transfected with Hs_*Ppargc1a*-Luc and increasing amounts of FLAG-TFEB (TFEB; 50, 100, 200, and 400 ng) as indicated or control (-) plasmid. Luciferase activity was normalized to expression of CMV-LacZ and expressed as fold increase. Data are represented as mean ± SD. Two-tailed *t*-test; ###$p<0.001$ vs. control transfection with pcDNA3.1; *$p<0.05$, **$p<0.01$, ***$p<0.001$ vs. transfection with TFEB only. (**B-D**) Luciferase assays performed with cell extracts of COS-7 cells transfected with Hs_*Ppargc1a*-Luc, FLAG-TFEB (TFEB) and increasing amounts (50, 100, 200, and 400 ng) of MYC-HDAC4 (HDAC4), MYC-HDAC5 (HDAC5), or MYC-HDAC7 (HDAC7) as indicated or control (-) plasmid. Luciferase activity was normalized to expression of CMV-LacZ and expressed as fold increase. Data are represented as mean±SD. One-way ANOVA q<0.0001 for (B); ###q<0.001 vs. control transfection with pcDNA3.1; *$q<0.05$; **$q<0.01$; ***$q<0.001$ vs. transfection with TFEB only. (**E-G**), COS-7 cells were transfected with expression plasmids encoding FLAG-TFEB, (E) HDAC4-MYC, (F) HDAC5-MYC, or (G) HDAC7-MYC, or constitutively active (ca) PKD1 (left panel), caPKD2 (middle panel), and caPKD3 (right panel) proteins, as indicated, together with the Hs_*Ppargc1a*-Luc reporter construct. Values were normalized to expression of CMV-LacZ and calculated as the fold increase in luciferase/CMV-LacZ ratio compared with the reporter alone. Data are represented as mean ± SD. One-way ANOVA q<0.0001 for (E-G); *$q<0.05$; **$q<0.01$; ***$q<0.001$. n=5.

Supplementary Figure 2. Deletion of TFEB leads to an increased expression of cardiac stress and remodeling markers and a decreased cardiac function. (**A**) Quantitative real-time polymerase chain reaction (qRT-PCR) analysis of *Tfeb*, *Tfe3* and *Mitf* expression from WT and cKO mice. mRNA expression was normalized to *Gapdh*. (**B**) Western blot analysis with anti-TFEB and anti-GAPDH antibodies (left; n.s.=nonspecific signal). GAPDH was used as loading control. Bar graph showing the ratio of the relative densities of TFEB and GAPDH protein contents. (**C**) Heart, lung and liver weights of WT and cKO mice normalized to tibia length. (**D**) Echocardiographic data for left ventricular ejection fraction (LVEF) and fractional shortening (FS) from WT and cKO mice. (**E**) Representative images of Hematoxylin and Eosin (H&E) stained heart cross-sections of WT and cKO mice. (**F**) Wheat Germ Agglutinin (WGA) stained histological cross-sections of hearts from WT and cKO mice (Scale bar, 100 µm) and (**G**) myocyte cross-sectional area (MCSA) measured on WGA stained sections with Image J. (**H**) Quantitative real-time polymerase chain reaction (qRT-PCR) analysis of indicated genes from WT and cKO mice. mRNA expression was normalized to *Gapdh*. (**I**) Western blot analysis with anti-β-MyHC and anti-GAPDH antibodies. GAPDH was used as loading control. Bar graph showing

the ratio of the relative densities of β-MyHC and GAPDH protein contents. (**J**) Representative images of Picrosirius Red stained (PSR, left) heart cross-sections of WT and cKO mice; scale bar, 1 mm. Fibrotic area (right) was measured with Image J. (**K**) qRT-PCR analysis of indicated genes from WT and cKO mice. mRNA expression was normalized to *Gapdh*. Data are presented as Mean±SD (WT: n=10, cKO: n=8; *q<0.05, **q<0.01, ***q<0.001, ****q<0.0001).

Supplementary Figure 3. Deletion of TFEB leads to an impaired regulation of genes involved in energy homeostasis. Quantitative real-time polymerase chain reaction (qRT-PCR) analysis of indicated metabolic genes (**A-B**) and genes involved in OXPHOS (**C**) and mitochondrial fission and fusion (**D**) from WT and cKO mice. mRNA expression was normalized to *Gapdh*. (**E**) Representative images of PAS stained heart cross-sections of WT and cKO mice; left: overview, scale bare 1mm; middle: higher magnification, scale bar 100 µm (corresponding to inset from left), right: quantification of glycogen content with Image J. (**A-E**) Data are presented as mean ± SD (WT: n=10, cKO: n=8; *q<0.05, **q<0.01, ***q<0.001, ****q<0.0001). (**F**) Volcano plots of differentially expressed genes in WT and cKO hearts. Genes that are significantly upregulated (red, q-value < 0.05), downregulated (blue, q-value < 0.05) or unchanged (gray, not significant (n.s.)) are indicated. The grid line on the y axis indicates the significant threshold of $-\log_{10}(q\text{-value}) = 1.3$.

Supplementary Figure 4. PO-induced cardiomyocyte hypertrophy is comparable between WT and cKO mice in the LVH model. (**A**) Quantitative real-time polymerase chain reaction (qRT-PCR) analysis of MiT/TFE genes in hearts of WT and cKO mice after 21 days of Sham or TAC surgery as indicated. mRNA expression was normalized to *Gapdh*. (**B**) Kinetics of left ventricular mass as determined by echocardiography at indicated time points after Sham and TAC surgery, respectively, of WT and cKO mice. (**C**) Representative images of Hematoxylin and Eosin (H&E) stained cardiac cross-sections of WT and cKO mice; scale bar, 100 µm. (**D-G**) Frequency-distribution histograms of myocyte cross-sectional areas (MCSA) measured with Image J on Wheat Germ Agglutinin (WGA) stained sections comparing WT_Sham and WT_TAC (D), cKO_Sham and cKO_TAC (E), WT_Sham and cKO_Sham (F), and WT_TAC and cKO_TAC (G). Data are presented as mean ± SD (21 days, WT Sham: n=10, WT TAC: n=13, cKO Sham: n=10, cKO TAC: n=15; *q<0.05, **q<0.01, ***q<0.001, ****q<0.0001).

Supplementary Figure 5. PO-induced cardiomyocyte hypertrophy is attenuated in cKO mice in the HFrEF model. (**A**) Quantitative real-time polymerase chain reaction (qRT-PCR) analysis of MiT/TFE genes in hearts of WT and cKO mice after 56 days of Sham or TAC surgery as indicated. mRNA expression was normalized to *Gapdh*. (**B**) Kinetics of left ventricular mass as determined by echocardiography at indicated time points after Sham and TAC surgery, respectively, of WT and cKO mice. (**C**) Representative images of Hematoxylin and Eosin (H&E) stained cardiac cross-sections of WT and cKO mice; scale bar, 100 µm. (**D-G**) Frequency-distribution histograms of myocyte cross-sectional areas (MCSA) measured with Image J on Wheat Germ Agglutinin (WGA) stained sections comparing WT_Sham and WT_TAC (D), cKO_Sham and cKO_TAC (E), WT_Sham and cKO_Sham (F), and WT_TAC and cKO_TAC (G). Data are presented as mean ± SD (56 days, WT Sham: n=10, WT TAC: n=6, cKO Sham: n=8, cKO TAC, n=6; *q<0.05, **q<0.01, ***q<0.001, ****q<0.0001).

Supplementary Figure 6. Proteomics data of WT and cKO mice after 56 days of Sham or TAC surgery. (**A**) Correlation between the cKO_TAC/cKO_Sham, (**B**) cKO_TAC/WT_TAC and (**C**) cKO_Sham/WT_Sham ratios and the WT_TAC/WT_Sham ratios. Ratio data is presented as single points. Correlation was calculated with Pearson r and p value. Simple linear regression is shown with 95% CI. Single protein analysis of (**D**) cardiac stress and remodeling markers and (**E**) proteins of the

energy metabolism, mitochondrial fission and fusion, and ETC. (56 days, WT Sham: n=10, WT TAC: n=6, cKO Sham: n=8, cKO TAC: n=6; *q<0.05, **q<0.01, ***q<0.001, ****q<0.0001).

Supplementary Figure 7. Analysis of proteomics data from hearts of WT and cKO mice after 56 days of Sham or TAC surgery. (**A**) Heatmaps of canonical metabolic pathways and (**B**) canonical signaling pathways with significant enrichment of regulated proteins are shown. Data are presented as heat maps (q-value ≤ 0.05, FC=1.3). Hatched marked boxes indicate that changes are not significant. (**C**) KEGG pathway of FAO related proteins (highlighted in red arrows) as shown in A.

1.2 Supplementary Tables

Supplementary Table 1: PCR primers for genotyping.

Name	Primer sequence
**Tcfeb_forward**	GTAGAACTGAGTCAAGGCATACTGG
**Tcfeb_reverse**	GGGTCCTACCTACCACAGAGCC
**αMHC-Cre_forward**	CGGCACTCTTAGCAAACCTC
**αMHC-Cre_reverse**	AGGCAAATTTTGGTGTACGG

Supplementary Table 2: qRT-PCR primers.

Name	Primer sequence
Acta1_forward	AGCCGGTGCTCTCCTACTG
Acta1_reverse	CGTCGCACATGGTGTCTAGT
Atp5a1_forward	CGGGCTGAGGAATGTTCA
Atp5a1_reverse	CCAAGTTCAGGGACATACCC
Col1a1_forward	TGTAAACACCCCAGCGAAGAA
Col1a1_reverse	CTGAGTTGCCATTTCCTTGGA
Col3a1_forward	CTCACCCTTCTTCATCCCACTCTTA
Col3a1_reverse	ACATGGTTCTGGCTTCCAGACAT
Cox4_forward	TCACTGCGCTCGTTCTGAT
Cox4_reverse	CGATCGAAAGTATGAGGGATG
Cpt1b_forward	GAGTGACTGGTGGGAAGAATATG
Cpt1b_reverse	GCTGCTTGCACATTTGTGTT
Drp1_forward	TCCCAATTCCATTATCCTCGC
Drp1_reverse	CATCAGTACCCGCATCCATG
Esrra_forward	GCCTCCAATGAGTGTGAGATC
Esrra_reverse	TTTGTACTTCTGCCGTCCG
Esrrb_forward	TGTGTTCCTCATCAACTGGG
Esrrb_reverse	CAGCTTGTCATCGTATGGGAG
Esrrg_forward	GGATGGGCAAAACATATTCCAG
Esrrg_reverse	GTTCATCCTCAAACGAAAGCG
Fis1_forward	AAGGGAGCAAAGAGGAACAG
Fis1_reverse	GCCCTCGCACATACTTTAGAG
Gys1_forward	CTGTCCTGTTCGGCTTCCT
Gys1_reverse	CCACATACGGCTTCTCTTCG
Hk2_forward	GCCAGCCTCTCCTGATTTTAGTGT
Hk2_reverse	GGGAACACAAAAGACCTCTTCTGG
Mfn1_forward	CATTGCGTTTCGGTTTTCCC
Mfn1_reverse	GAAGGAGCAGTAGGAGTTGAAG
Mfn2_forward	CGAGGCTCTGGATTCACTTC
Mfn2_reverse	CAACCAGCCAGCTTTATTCC
Mitf_forward	AAAAGTCAACCTCTGAAGAGCAG
Mitf_reverse	GCGTAGCAAGATGCGTGAT
MT-Co1_forward	TCCACTATTTGTCTGATCCGTACT
MT-Co1_reverse	AGTAGTATAGTAATGCCTGCGGCTA
MT-Cytb_forward	CATTTATTATCGCGGCCCTA
MT-Cytb_reverse	TGGGTTGTTTGATCCTGTTTC
MT-Nd1_forward	CTAGCAGAAACAAACCGGGC
MT-Nd1_reverse	CCGGCTGCGTATTCTACGTT
MT-Nd4_forward	CCAAACTCCATGAAGCTTCATAGG

MT-Nd4_reverse	GATGATGTGAGGCCATGTGCG
Myh6_forward	GCCAAGACTGTCCGGAATGA
Myh6_reverse	TGGAAGATCACCCGGGACTT
Myh7_forward	CGCATCAAGGAGCTCACC
Myh7_reverse	CTGCAGCCGCAGTAGGTT
Ndufb8_forward	TCCCTTCCTACCAGCCTGT
Ndufb8_reverse	GAGCAGGAAAACAGGAATGC
Nppa_forward	GGGGGTAGGATTGACAGGAT
Nppa_reverse	ACACACCACAAGGGCTTAGG
Nppb_forward	GCACAAGATAGACCGGATCG
Nppb_reverse	CTTCAAAGGTGGTCCCAGAG
Nrf1_forward	TGGAGTCCAAGATGCTAATGG
Nrf1_reverse	GCGAGGCTGGTTACCACA
Ppara_forward	CTGAGACCCTCGGGGAAC
Ppara_reverse	AAACGTCAGTTCACAGGGAAG
Ppargc1a_forward	TGAAAGGGCCAAACAGAGAG
Ppargc1a_reverse	GTAAATCACACGGCGCTCTT
Pppargc1b_forward	CCACAGCCCACTTCCAGA
Pppargc1b_reverse	CCAAGAGAGTCGCTTTGTGA
Sdha_forward	CCCTGAGCATTGCAGAATC
Sdha_reverse	TCTTCTCCAGCATTTGCCTTA
Sdhb_forward	ACTGGTGGAACGGAGACAAG
Sdhb_reverse	CCTCTGTGAAGTCGTCTCTGG
Slc2a1_forward	TTGTTGTAGAGCGAGCTGGA
Slc2a1_reverse	TTCAAAGAAGGCCACAAAGC
Slc2a4_forward	GTCGGGTTTCCAGCAGAT
Slc2a4_reverse	GGCATTGATAACCCCAATGT
Tfam_forward	CAAAGGATGATTCGGCTCAG
Tfam_reverse	AAGCTGAATATATGCCTGCTTTTC
Tfeb_forward	GAGCTGGGAATGCTGATCC
Tfeb_reverse	CTTGAGGATGGTGCCTTTGT
Tfe3_forward	GCTCAAAAGCCAACCCCTAT
Tfe3_reverse	GGTGTGGCCTGCAGTGATA
Uqcrc2_forward	TGGGCTCTTTGGAATTTACAC
Uqcrc2_reverse	TTGGTTGTAGGCAGCATTGA

Supplementary Table 3: LC-MS/MS parameters (A) and Spectronaut parameters for peptide/Protein identification and intensity extraction (B).

Supplementary Table 3A: LC-MS/MS parameters (data independent mode; quantitative data).

**Data independent acquisition (DIA)**	
**Reversed phase liquid chromatography**	**Ultimate 3000 RSLC (Thermo Scientific)**
Trap column	75 µm inner diameter, packed with 3 µm C18 particles (Acclaim PepMap100, Thermo Scientific)
Analytical column	75 µm inner diameter, packed with 2.6 µm C18 particles (Accucore, 25 cm, Thermo Scientific)
Flow rate	300 nl/min
Column oven temperature	40°C
Buffer system	binary buffer system consisting of 0.1% acetic acid in HPLC-grade water (buffer A) and 100% ACN in 0.1% acetic acid (buffer B)
Gradient	gradient of buffer B: 2min 2% to 5 %, 8min 7%, 60min 7% to 25%, 5min 25 to 40%, 2 min 40% to 90%, 6 min 90%, 2 min 90% to 2%, 10 min 2%
**Mass spectrometer**	Exploris 480
Operation mode	Data-independent
Electrospray	Nanospray Flex Ion Source
**Full MS**	
MS scan resolution	120,000
Normalized AGC target	300%
Maximum ion injection time for the MS scan	60 ms
Scan range	350 to 1,200 m/z
Spectra data type	profile
**dd-MS2**	
Resolution	30,000
Normalized MS/MS AGC target	3,000
Maximum ion injection time for the MS/MS scans	auto
Spectra data type	profile
Selection for MS/MS	1
Isolation window	65 windows m/z 13 (overlap m/z 2)
Fixed first mass	200
Dissociation mode	higher energy collisional dissociation (HCD)
Normalized collision energy	fixed, 30
Dissociation mode	HCD

Supplementary Table 3B: Spectronaut parameters for peptide/Protein identification and intensity extraction.

**Spectronaut 14.10**	
Computer Name: AGVOE-SPECTRONA	
User Domain Name: AGVOE-SPECTRONA	
User Name: spectronaut	
Analysis Mode: UI	
Analysis Type: directDIA	
Analysis Date: 20-April-2021	
Settings Used:	
**Pulsar Search\Peptides**	
Toggle N-terminal M:	True
Min Peptide Length:	7
Max Peptide Length:	52
Missed Cleavages:	2
Digest Type:	Specific
Enzymes / Cleavage Rules:	Trypsin/P
**Pulsar Search\Labeling**	
Channels:	
Channel 1:	False
Channel 2:	False
Channel 3:	False
**DIA Analysis\Data Extraction**	
MS1 Mass Tolerance Strategy:	Dynamic
Correction Factor:	1
MS2 Mass Tolerance Strategy:	Dynamic
Correction Factor:	1
Intensity Extraction MS1:	Maximum Intensity
Intensity Extraction MS2:	Maximum Intensity
**DIA Analysis\XIC Extraction**	
XIC IM Extraction Window:	Dynamic
Correction Factor:	1
XIC RT Extraction Window:	Dynamic
Correction Factor:	1
**Pulsar Search\Modifications**	
Max Variable Modifications:	5
**Database**	
Original File:	Uniprot_2_2021.fasta (Mus musculus)
**Select Modifications:**	
Fixed Modifications:	Carbamidomethyl (C)
Variable Modifications:	Acetyl (Protein N-term), Oxidation (M)

DIA Analysis\Calibration

MS1 Mass Tolerance Strategy:	System Default
MS2 Mass Tolerance Strategy:	System Default
Precision iRT:	True
iRT <-> RT Regression Type:	Local (Non-Linear) Regression
Exclude Deamidated Peptides:	True
MZ Extraction Strategy:	Maximum Intensity
Allow source specific iRT Calibration:	True

DIA Analysis\Identification

Generate Decoys:	True
Decoy Limit Strategy:	Dynamic
Library Size Fraction:	0.1
Decoy Method:	Mutated
Preferred Fragment Source:	NN Predicted Fragments
Machine Learning:	Per Run
Exclude Duplicate Assays:	True
Precursor PEP Cutoff:	1
Protein Qvalue Cutoff (Experiment):	0.01
Protein Qvalue Cutoff (Run):	0.05
Exclude Single Hit Proteins:	False
Pvalue Estimator:	Kernel Density Estimator
Precursor Qvalue Cutoff:	0.001
Single Hit Definition:	By Stripped Sequence

DIA Analysis\Quantification

Interference Correction:	True
MS1 Min:	2
MS2 Min:	3
Exclude All Multi-Channel Interferences:	True
Only Identified Peptides:	True
Protein LFQ Method:	Automatic
Major (Protein) Grouping:	by Protein Group Id
Minor (Peptide) Grouping:	by Stripped Sequence
Minor Group Top N:	False
Minor Group Quantity:	Sum precursor quantity
Major Group Top N:	True
Min:	2
Max:	3
Major Group Quantity:	Mean peptide quantity
Quantity MS-Level:	MS2
Quantity Type:	Area
Proteotypicity Filter:	None
Data Filtering:	Qvalue percentile
Fraction:	0.25
Imputing Strategy:	No Imputing

Cross Run Normalization:	False
**DIA Analysis\PTM Workflow**	
PTM Localization:	True
Probability Cutoff:	0.75
PTM Analysis:	True
Multiplicity:	True
Run Clustering:	False
PTM Consolidation:	Sum
Flanking Region:	7
**DIA Analysis\Workflow**	
MS2 DeMultiplexing:	Automatic
Run Limit for directDIA Library:	-1
Method Evaluation:	False
Profiling Strategy:	iRT Profiling
Profiling Row Selection:	Minimum Qvalue Row Selection
Qvalue Threshold:	0.001
Profiling Target Selection:	Profile only non-identfied Precursor
Identification Criterion:	Qvalue
Threshold:	0.001
Carry-over exact Peak Boundaries:	False
Unify Peptide Peaks Strategy:	Select corresponding Peak
**DIA Analysis\Protein Inference**	
Protein Inference Workflow:	Automatic
Inference Algorithm:	IDPicker
**DIA Analysis\Post Analysis**	
Calculate Sample Correlation Matrix:	True
Calculate Explained TIC:	Quick
Differential Abundance Grouping:	Major Group (Quantification Settings)
Smallest Quantitative Unit:	Precursor Ion (Quantification Settings)
Use All MS-Level Quantities:	False
Differential Abundance Testing:	Paired *t*-test
Group-Wise Testing Correction:	False
Run Clustering:	True
Distance Metric:	Manhattan Distance
Linkage Strategy:	Ward's Method
Z-score transformation:	False
Order Runs by Clustering:	True
**DIA Analysis\Pipeline Mode**	
Post Analysis Reports:	
Scoring Histograms:	True
Data Completeness Bar Chart:	True
Run Identifications Bar Chart:	True
CV Density Line Chart:	True

CVs Below X Bar Chart:	True
Generate SNE File:	True
Store Iontraces in SNE:	False
Report Schema:	C_FunGene_complex (Normal)
Reporting Unit:	Across Experiment
**Pulsar Search\Identification**	
Peptide FDR:	0.01
Protein Group FDR:	0.01
PSM FDR:	0.01
**Pulsar Search\Tolerances**	
Tolerance Parameters:	
Thermo Orbitrap:	
Calibration Search:	Dynamic
MS1 Correction Factor:	1
MS2 Correction Factor:	1
Main Search:	Dynamic
MS1 Correction Factor:	1
MS2 Correction Factor:	1
TOF:	
Calibration Search:	Dynamic
MS1 Correction Factor:	1
MS2 Correction Factor:	1
Main Search:	Dynamic
MS1 Correction Factor:	1
MS2 Correction Factor:	1
Thermo IonTrap:	
Calibration Search:	Dynamic
MS1 Correction Factor:	1
MS2 Correction Factor:	1
Main Search:	Dynamic
MS1 Correction Factor:	1
MS2 Correction Factor:	1
**Pulsar Search\Workflow**	
Use DNN Predicted Ion Mobility:	Auto
Fragment Ion Selection Strategy:	Intensity Based
In-Silico Generate Missing Channels:	False
**Pulsar Search\Result Filters**	
Precursors:	
Best N Fragments per Peptide:	True
Min:	6
Max:	10
Channel Count:	False
Modifications:	None
Amino Acids:	False

Best N Peptides per Protein Group:	False
FASTA Matched:	False
Missed Cleavage:	False
Peptide Charge:	False
Proteotypicity:	False
Fragment Ions:	
m/z:	True
Min:	300
Max:	1,800
Ion Charge:	False
Ion Loss Type:	False
Ion Type:	False
Ion AA Length:	True
N:	3
Relative Intensity:	True
Min:	5

Supplementary Table 4: Subunits of the electron transport chain.

Complex	Gene	Gen name
**Complex I**	*MT-Nd1*	NADH-ubiquinone oxidoreductase chain 1
	MT-Nd4	NADH-ubiquinone oxidoreductase chain 4
	Ndufb8	NADH dehydrogenase [ubiquinone] 1 beta subcomplex subunit 8, mitochondrial
**Complex II**	*Sdha*	Succinate dehydrogenase [ubiquinone] flavoprotein subunit, mitochondrial
	Sdhb	Succinate dehydrogenase [ubiquinone] iron-sulfur subunit, mitochondrial
**Complex III**	*MT-Cytb*	Cytochrome b
	Uqcrc2	Cytochrome b-c1 complex subunit 2, mitochondrial
**Complex IV**	*Cox4*	Cytochrome c oxidase subunit 4 isoform 1, mitochondrial
	MT-Co1	Cytochrome c oxidase subunit 1
**Complex V**	*Atp5a1*	ATP synthase subunit alpha, mitochondrial

Supplementary Table 5. Summarized echocardiography data from WT and cKO mice after 21 days of TAC.

Attached as “Table S5 - Echo - 21 days of TAC – eng.xlsx”

Supplementary Table 6. Summarized echocardiography data from WT and cKO mice after 56 days of TAC.

Supplementary Table 6A. WT_TAC vs. WT_Sham & cKO_TAC vs. cKO_Sham.

Supplementary Table 6B. cKO_Sham vs. WT_Sham & cKO_TAC vs. WT_TAC.

Attached as “Table S6 A,B - Echo - 56 days of TAC - eng.xlsx”
