# Supplementary material for: Metabolic remodeling in cardiac hypertrophy and heart failure with reduced ejection fraction occurs independent of transcription factor EB in mice
Source: Front Cardiovasc Med. 2024 Jan 8;10:1323760. doi: 10.3389/fcvm.2023.1323760 (PMC10800928; doi:10.3389/fcvm.2023.1323760)
Supplement: Supplementary file 4 [file Datasheet2.pdf]

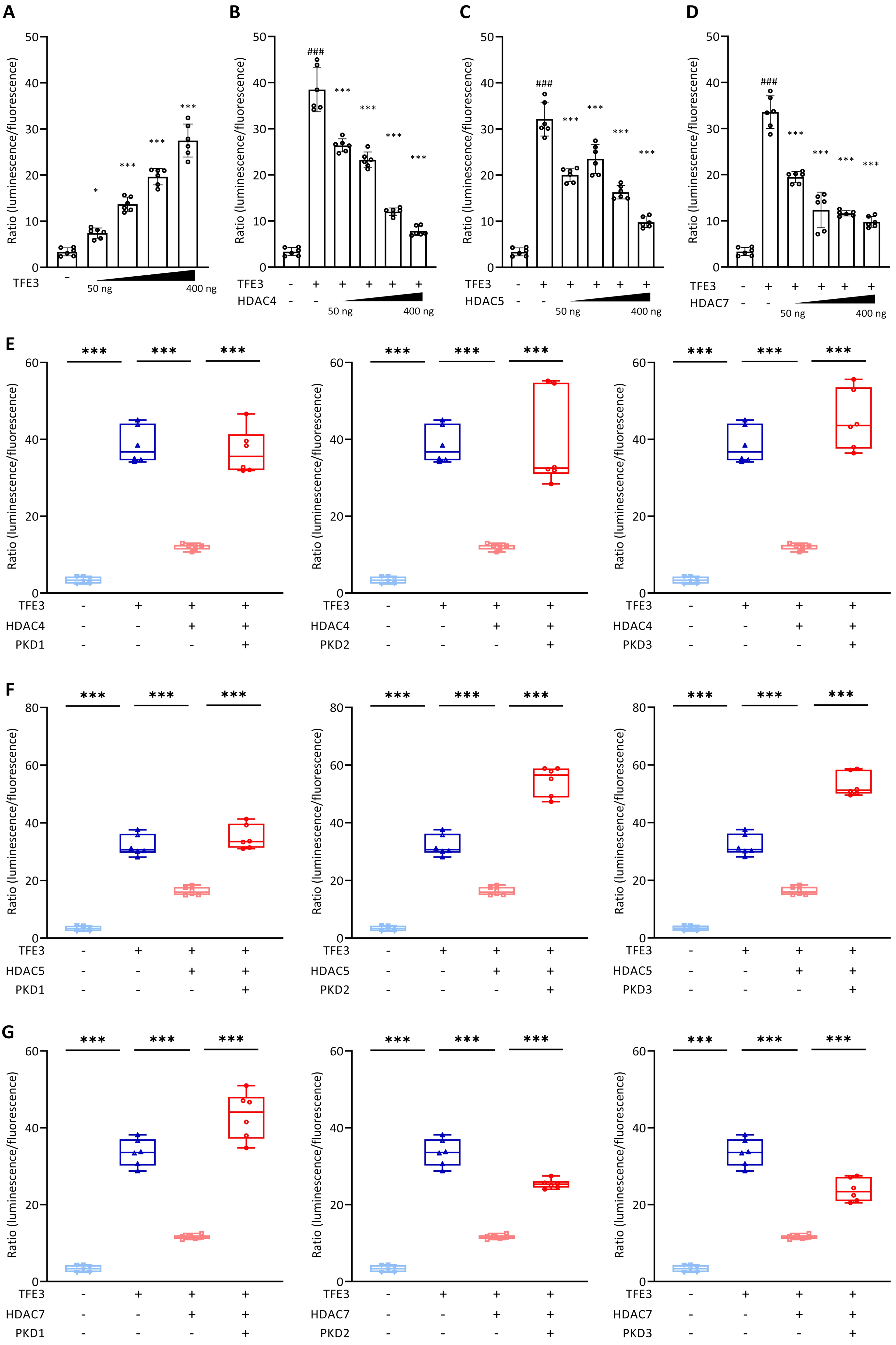

A
B
C
D
E
F
G
Ratio (luminescence/fluorescence)
TFE3
HDAC4
HDAC5
HDAC7
PKD1
PKD2
PKD3
50 ng
400 ng
###
***


Figure S1

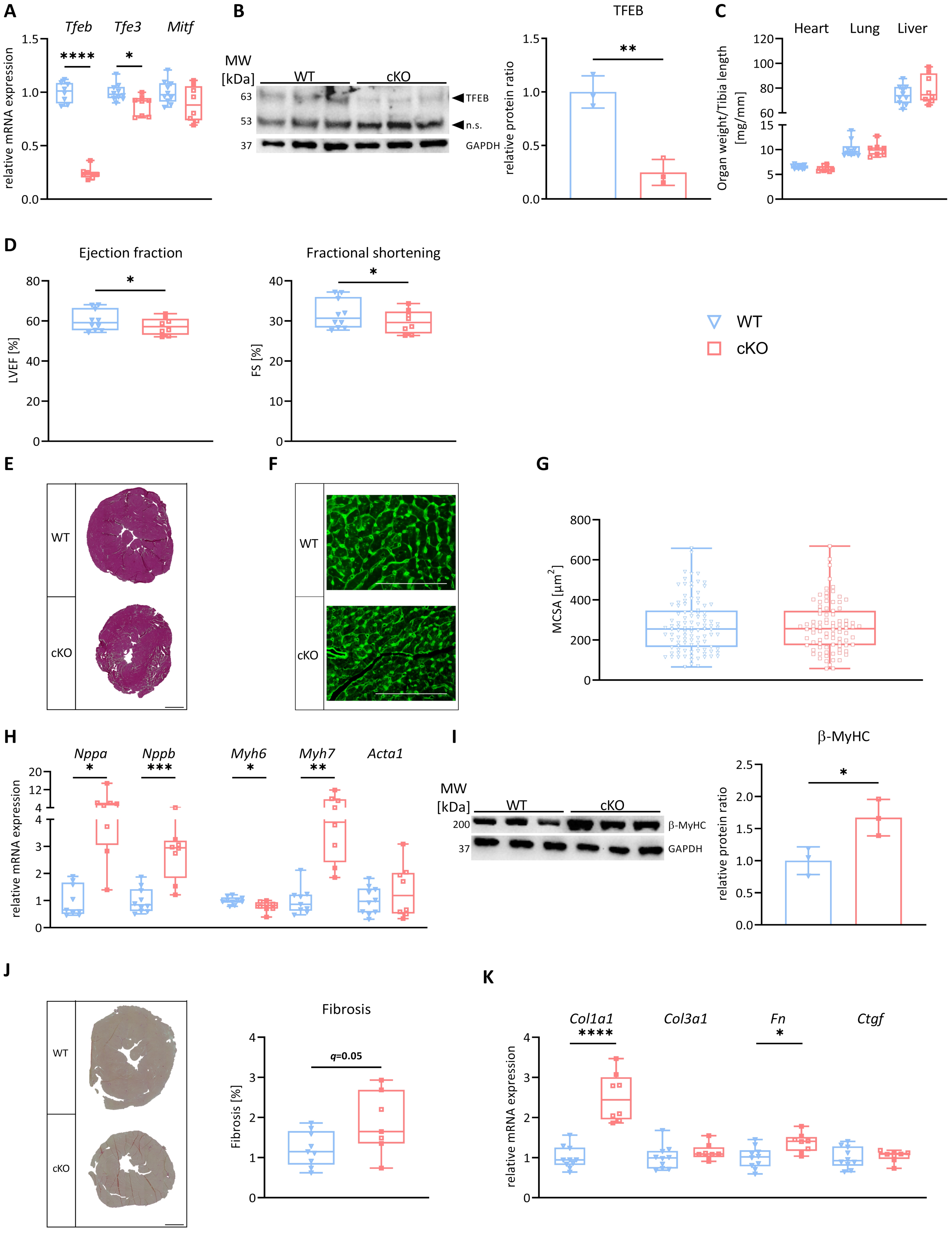
A
Tfeb
Tfe3
Mitf
relative mRNA expression
****
*
B
MW [kDa]
WT
cKO
63
53
37
TFEB
n.s.
GAPDH
relative protein ratio
**
C
Heart
Lung
Liver
Organ weight/Tibia length [mg/mm]
D
Ejection fraction
LVEF [%]
*
Fractional shortening
FS [%]
*
WT
cKO
E
F
G
MCSA [µm2]
H
Nppa
Nppb
Myh6
Myh7
Acta1
***
**
I
β-MyHC
200
GAPDH
J
Fibrosis
Fibrosis [%]
q=0.05
K
Col1a1
Col3a1
Fn
Ctgf

Figure S2

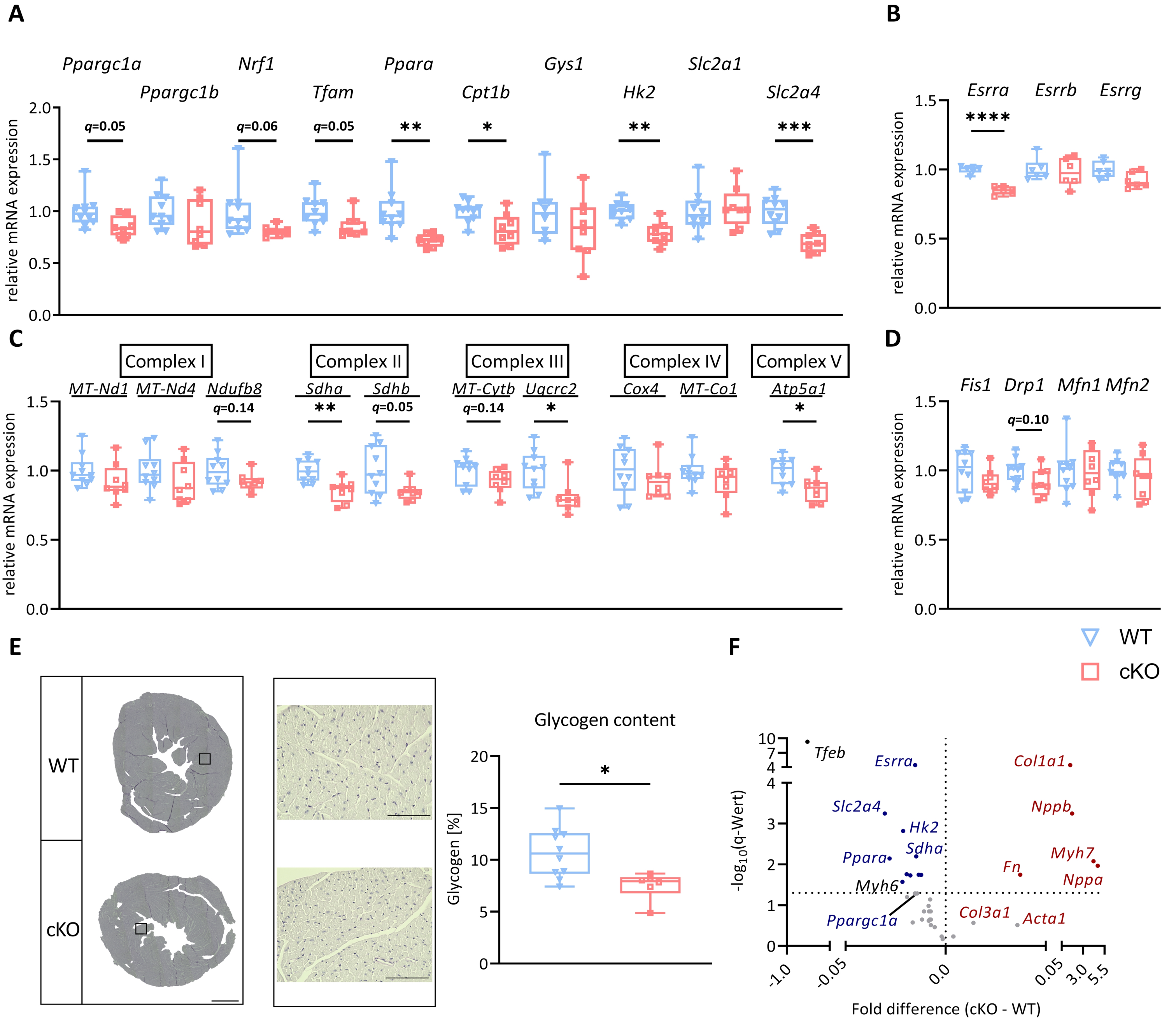

A
Ppargc1a
Ppargc1b
Nrf1
Tfam
Ppara
Cpt1b
Gys1
Hk2
Slc2a1
Slc2a4
q=0.05
q=0.06
q=0.05
relative mRNA expression
B
Esrra
Esrrb
Esrrg
C
Complex I
Complex II
Complex III
Complex IV
Complex V
MT-Nd1
MT-Nd4
Ndufb8
Sdha
Sdhb
MT-Cytb
Uqcrc2
Cox4
MT-Co1
Atp5a1
q=0.14
q=0.05
q=0.14
D
Fis1
Drp1
Mfn1
Mfn2
q=0.10
WT
cKO
E
WT
cKO
Glycogen content
Glycogen [%]
F
-log10(q-Wert)
Tfeb
Esrra
Col1a1
Slc2a4
Hk2
Nppb
Sdha
Ppara
Myh7
Fn
Nppa
Myh6
Col3a1
Acta1
Ppargc1a
Fold difference (cKO - WT)


**Figure S3**

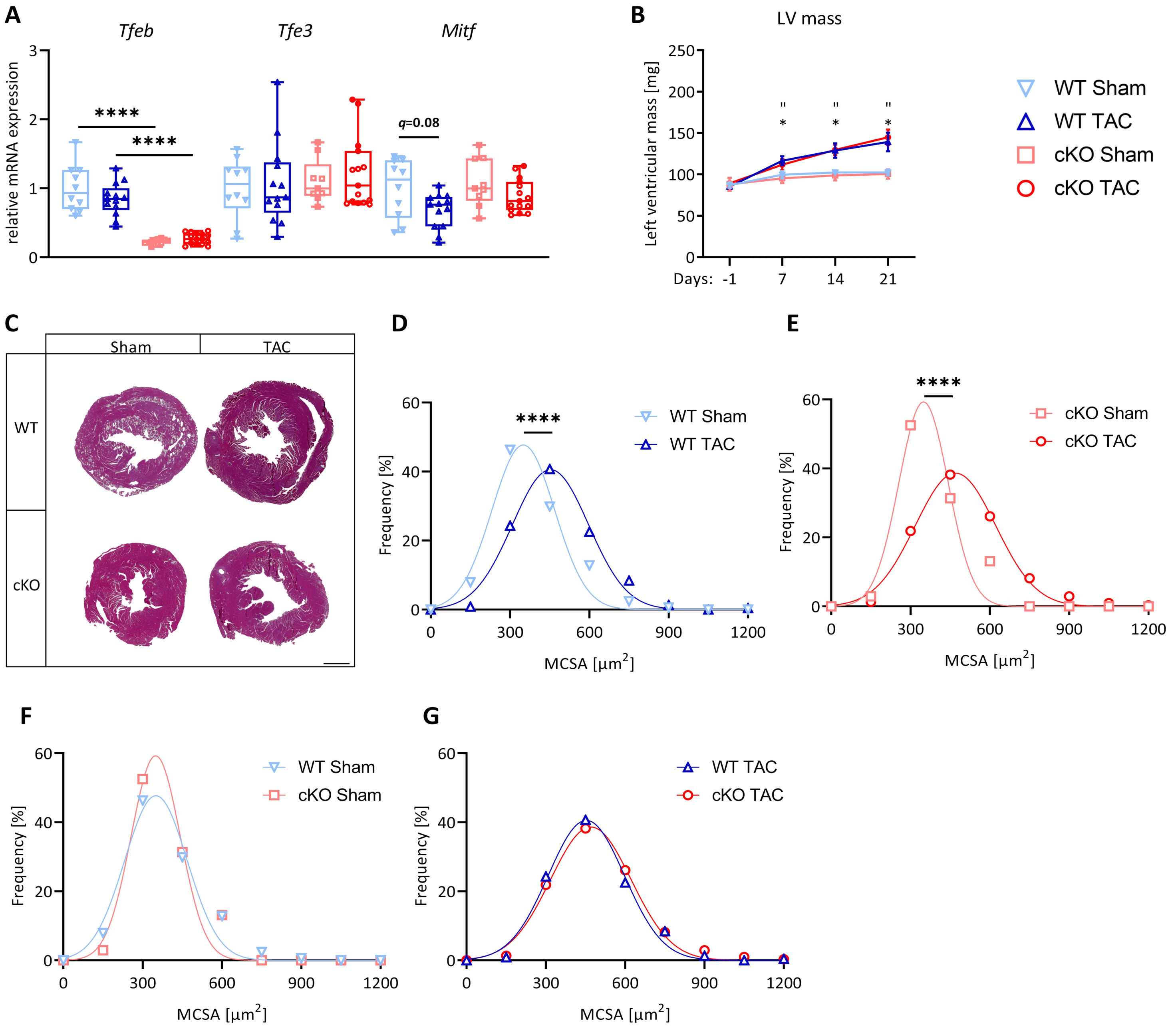
A
Tfeb
Tfe3
Mitf
relative mRNA expression
****
****
q=0.08
B
LV mass
Left ventricular mass [mg]
Days:
WT Sham
WT TAC
cKO Sham
cKO TAC
C
Sham
TAC
WT
cKO
D
E
Frequency [%]
MCSA [μm2]
****
F
G

**Figure S4**

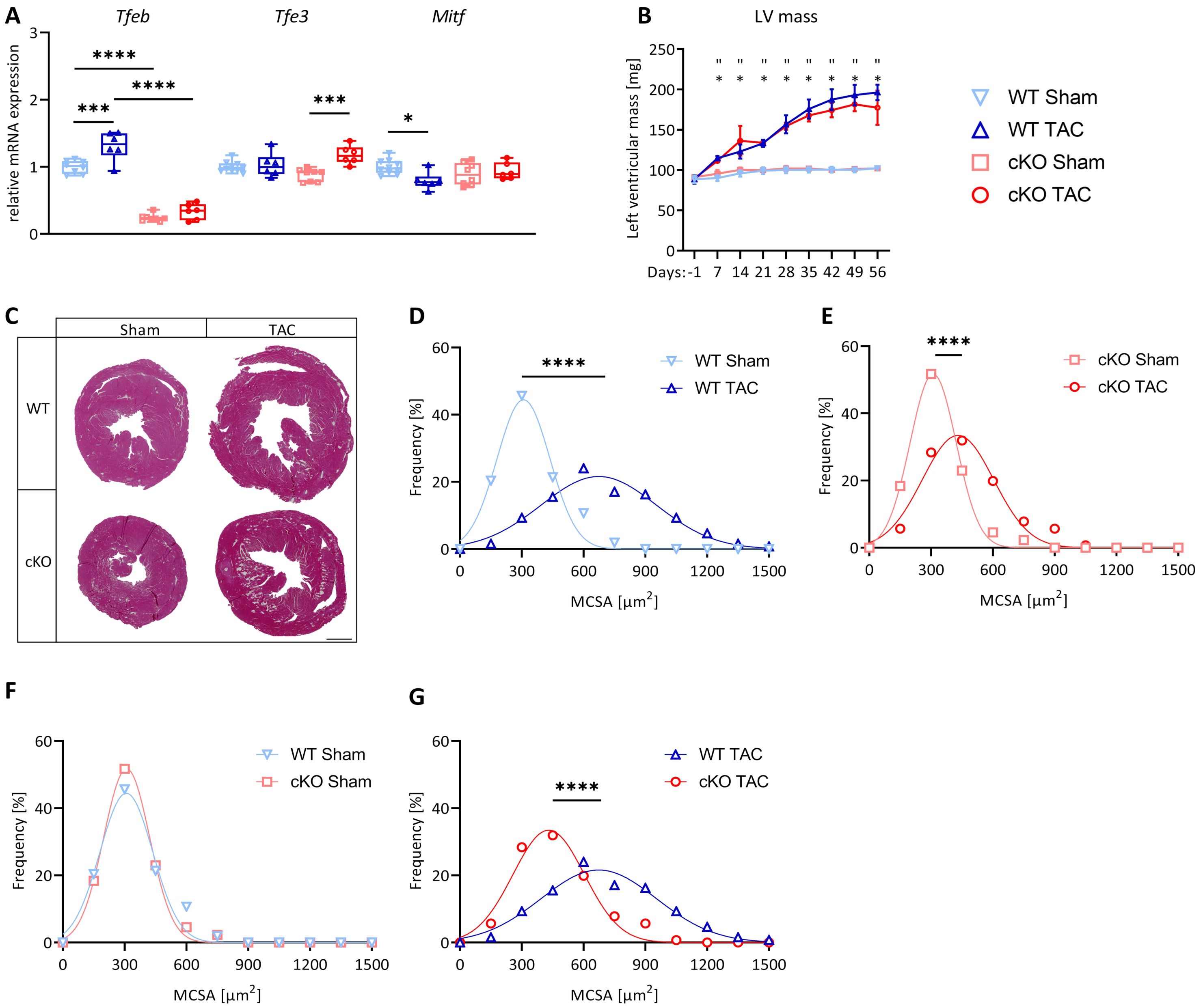

A
Tfeb
Tfe3
Mitf
relative mRNA expression
****
***
****
***
*
B
LV mass
Left ventricular mass [mg]
Days: -1 7 14 21 28 35 42 49 56
WT Sham
WT TAC
cKO Sham
cKO TAC
C
Sham
TAC
WT
cKO
D
Frequency [%]
MCSA [μm2]
E
F
G


**Figure S5**

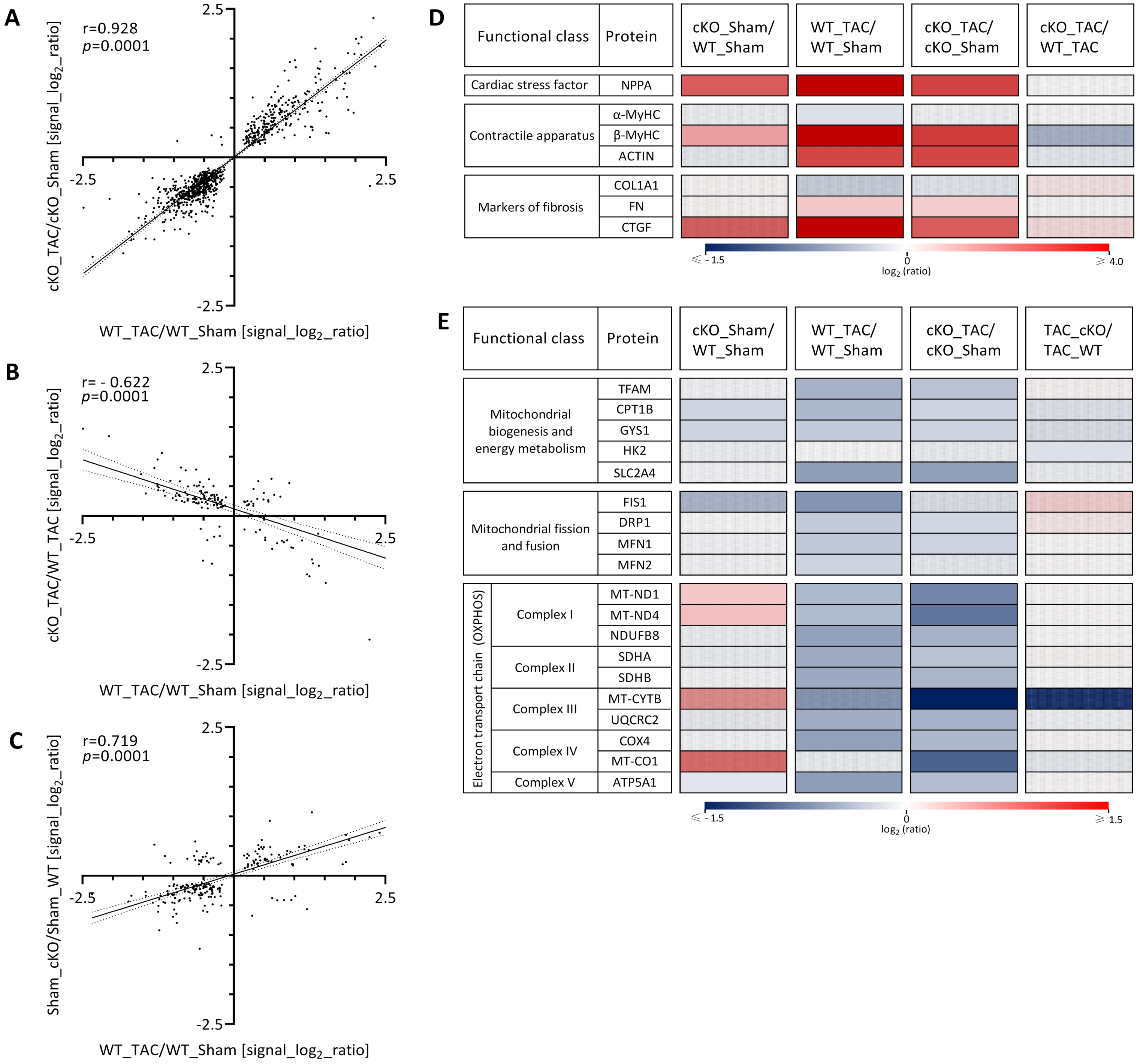
A
r=0.928
p=0.0001
cKO_TAC/cKO_Sham [signal_log2_ratio]
WT_TAC/WT_Sham [signal_log2_ratio]
2.5
-2.5
B
r= - 0.622
p=0.0001
cKO_TAC/WT_TAC [signal_log2_ratio]
WT_TAC/WT_Sham [signal_log2_ratio]
C
r=0.719
p=0.0001
Sham_cKO/Sham_WT [signal_log2_ratio]
WT_TAC/WT_Sham [signal_log2_ratio]
D
Functional class
Protein
cKO_Sham/ WT_Sham
WT_TAC/ WT_Sham
cKO_TAC/ cKO_Sham
cKO_TAC/ WT_TAC
Cardiac stress factor
NPPA
Contractile apparatus
α-MyHC
β-MyHC
ACTIN
Markers of fibrosis
COL1A1
FN
CTGF
⩽ - 1.5
0
⩾ 4.0
log2 (ratio)
E
Functional class
Protein
cKO_Sham/ WT_Sham
WT_TAC/ WT_Sham
cKO_TAC/ cKO_Sham
TAC_cKO/ TAC_WT
Mitochondrial biogenesis and energy metabolism
TFAM
CPT1B
GYS1
HK2
SLC2A4
Mitochondrial fission and fusion
FIS1
DRP1
MFN1
MFN2
Electron transport chain (OXPHOS)
Complex I
MT-ND1
MT-ND4
NDUFB8
Complex II
SDHA
SDHB
Complex III
MT-CYTB
UQCRC2
Complex IV
COX4
MT-CO1
Complex V
ATP5A1
⩽ - 1.5
0
⩾ 1.5
log2 (ratio)

**Figure S6**

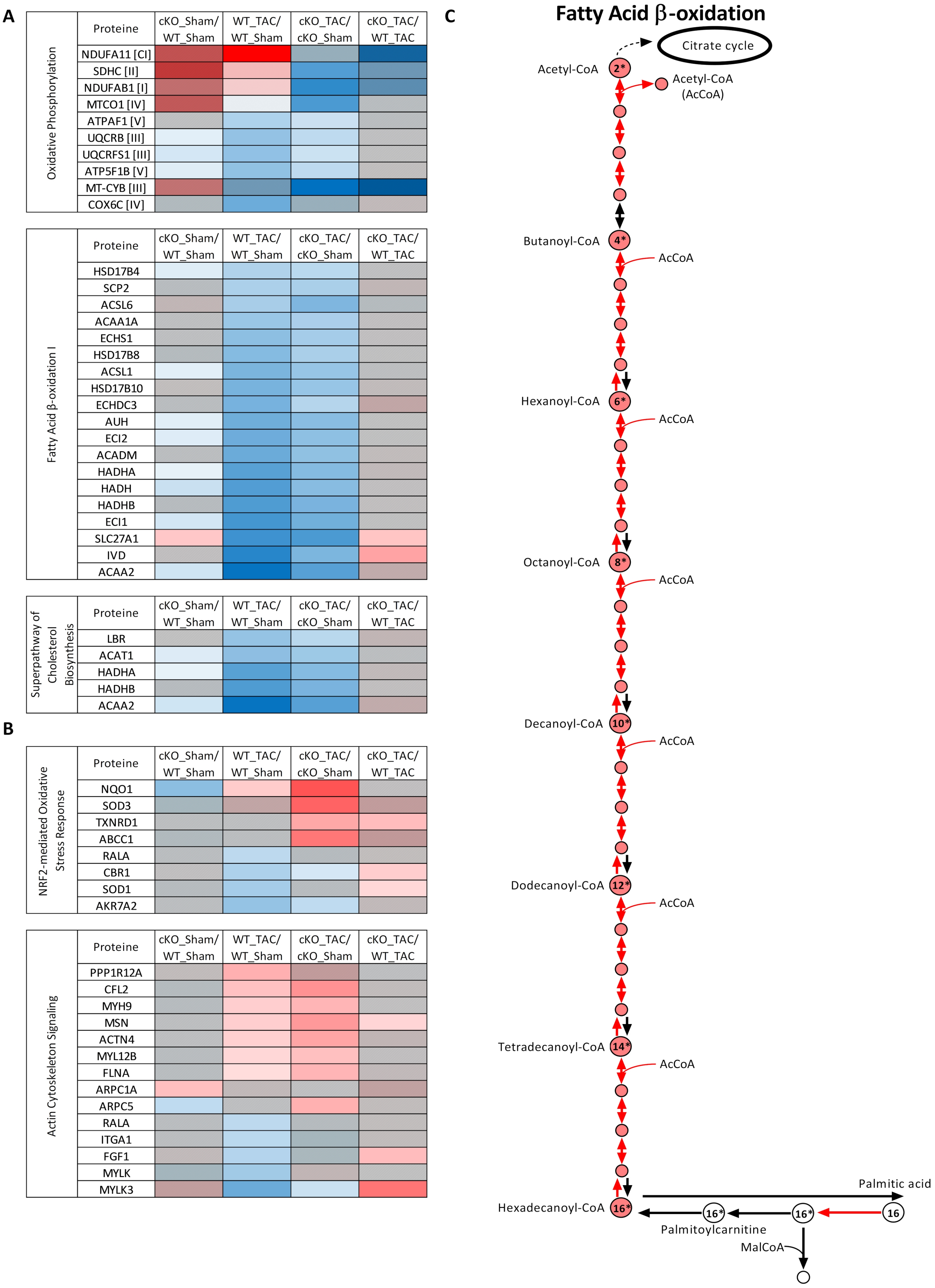
A
Oxidative Phosphorylation
Proteine
cKO_Sham/ WT_Sham
WT_TAC/ WT_Sham
cKO_TAC/ cKO_Sham
cKO_TAC/ WT_TAC
NDUFA11 [CI]
SDHC [II]
NDUFAB1 [I]
MTCO1 [IV]
ATPAF1 [V]
UQCRB [III]
UQCRFS1 [III]
ATP5F1B [V]
MT-CYB [III]
COX6C [IV]
Fatty Acid β-oxidation I
HSD17B4
SCP2
ACSL6
ACAA1A
ECHS1
HSD17B8
ACSL1
HSD17B10
ECHDC3
AUH
ECI2
ACADM
HADHA
HADH
HADHB
ECI1
SLC27A1
IVD
ACAA2
Superpathway of Cholesterol Biosynthesis
LBR
ACAT1
HADHA
HADHB
ACAA2
B
NRF2-mediated Oxidative Stress Response
NQO1
SOD3
TXNRD1
ABCC1
RALA
CBR1
SOD1
AKR7A2
Actin Cytoskeleton Signaling
PPP1R12A
CFL2
MYH9
MSN
ACTN4
MYL12B
FLNA
ARPC1A
ARPC5
RALA
ITGA1
FGF1
MYLK
MYLK3
C
Fatty Acid β-oxidation
Citrate cycle
Acetyl-CoA
2*
Acetyl-CoA (AcCoA)
Butanoyl-CoA
4*
AcCoA
Hexanoyl-CoA
6*
AcCoA
Octanoyl-CoA
8*
AcCoA
Decanoyl-CoA
10*
AcCoA
Dodecanoyl-CoA
12*
AcCoA
Tetradecanoyl-CoA
14*
AcCoA
Hexadecanoyl-CoA
16*
Palmitic acid
16*
16*
16
Palmitoylcarnitine
MalCoA

**Figure S7**
